# Peptide-based antimicrobial effect against carbapenem-resistant *Acinetobacter baumannii*: preclinical drug assessment and translational potential

**DOI:** 10.3389/fphar.2026.1732644

**Published:** 2026-02-18

**Authors:** Hak Jun Lee, Seung Jun Lee, Yoon Hyun Sung, Seung Min Park, Seung Pyo Choi, Yangmee Kim, Young Kyung Yoon

**Affiliations:** 1 Institute of Emerging Infectious Diseases, Korea University, Seoul, Republic of Korea; 2 R&D Team, HLB Science, Seoul, Republic of Korea; 3 Department of Bioscience and Biotechnology, Konkuk University, Seoul, Republic of Korea; 4 Division of Infectious Diseases, Department of Internal Medicine, Korea University College of Medicine, Seoul, Republic of Korea

**Keywords:** *Acinetobacter* baumannii, animal model, antimicrobial peptides, carbapenem-resistant, combination therapy

## Abstract

**Background:**

Carbapenem-resistant *Acinetobacter baumannii* (CRAB) has become a major global public health threat, with limited treatment options and poor clinical outcomes. In this study, we evaluated the potential of antimicrobial peptides as adjuvants in the treatment of CRAB.

**Methods:**

*In vitro* synergistic and bactericidal effects of DD-S052P in combination with the US Food and Drug Administration-approved antibiotics against CRAB were assessed using the checkerboard method and time-kill assay. Mice infected with CRAB were used to evaluate the survival-enhancing effects of DD-S052P-based combination therapy. CRAB isolates were obtained from blood samples of patients with bacteremia.

**Results:**

In both the checkerboard method and time-kill assay, DD-S052P demonstrated *in vitro* synergistic activity against CRAB isolates when combined with ampicillin/sulbactam, ceftolozane/tazobactam, and colistin. Notably, all combinations exhibited bactericidal effects against CRAB isolates in the time-kill assay. Combination therapy with DD-S052P plus colistin yielded a 100% survival rate in the mouse infection models.

**Conclusion:**

Our findings indicate that DD-S052P in combination with colistin may represent a promising therapeutic option for CRAB infections. Further research is required to elucidate the mechanisms by which DD-S052P overcomes existing carbapenem resistance and validate its clinical applicability.

## 1 Introduction

Antimicrobial resistance poses a critical threat to global public health and is recognized as a silent pandemic, contributing to 4.95 million deaths in 2019 (Antimicrobial Resistance Collaborators, 2022). In particular, current treatment options for infections caused by carbapenem-resistant *Acinetobacter baumannii* (CRAB) remain limited and are associated with poor clinical outcomes and notable pharmacokinetic limitations, including high toxicity and low plasma levels (Zhang et al., 2024). Among innovative approaches, synergistic therapeutic strategies combining antibiotics and antimicrobial peptides have shown potential in combating CRAB infections (Park et al., 2016; Wu et al., 2017; Jahangiri et al., 2021).

In this context, antimicrobial peptides have emerged as potential alternative or adjunctive therapeutic agents to conventional antibiotics. They exhibit a broad spectrum of antimicrobial activity, target multiple sites of action, have a low potential for drug-induced resistance, possess anti-biofilm properties, demonstrate high efficacy at low doses, and exert immunomodulatory properties (Isler et al., 2019; Marr et al., 2006).

*Mycobacterium tuberculosis* adenylate kinase (ADK), with a molecular weight of 20 kDa, is a globular protein that encompasses a catalytic core composed of an α/β domain and two flexible lid domains—namely, the ATP-lid and the AMP-binding domain (Bellinzoni et al., 2006). The recombinant *M. tuberculosis* ADK protein was found to bind *Escherichia coli* lipopolysaccharide (LPS), suggesting that it may possess LPS-binding properties (Lee et al., 2025). DD-S052P was *de novo* designed based on the amino acid residues from Lys44 to Asp54 in the AMP-binding domain of *M. tuberculosis* ADK (Lee et al., 2025). It is a synthetic linear 11-amino acid peptide with enhanced stability conferred by modifications (H-AEEAc-klrvklrrylr-NH2), including 2-(2-(2-aminoethoxy) ethoxy) acetic acid at the N-terminus (Supplementary Figure S1A). All amino acid residues of DD-S052P are D-amino acids, enhancing its resistance to proteolysis (Supplementary Figure S1A). Isothermal titration calorimetry demonstrated that DD-S052P binds to LPS with a micromolar affinity, exhibiting a dissociation constant (K_D_) of 2.8 × 10^−6^ M (Supplementary Figure S1B). Scanning electron microscopy revealed that DD-S052P-treated *E*. *coli* exhibited a roughened membrane surface, suggesting that DD-S052P exerts bactericidal activity through membrane disruption (Supplementary Figure S1C). Circular dichroism (CD) experiments demonstrated that DD-S052P adopts a random-coil structure in aqueous solution but transitions to an α-helical structure in dodecylphosphocholine micelles, which mimics the bacterial membrane environment (Supplementary Figure S1D).

In this study, we aimed to evaluate the potent antimicrobial activity of DD-S052P against CRAB isolates and determine the most effective combination of DD-S052P with conventional antibiotics. These combinations may produce synergistic effects that surpass the efficacy of individual agents, highlighting a promising strategy to combat multidrug-resistant (MDR) infections.

## 2 Materials and methods

### 2.1 Peptide and chemical reagents

The antimicrobial candidate DD-S052P was produced by Bachem AG (Bubendorf, Switzerland) at the request of HLB Science Inc. using solid-phase peptide synthesis. The peptide was cleaved from the resin with full side-chain deprotection, followed by purification using preparative high-performance liquid chromatography (HPLC), ion exchange treatment, sterile microfiltration, and lyophilization. The final product had a purity of ≥95.0%. Its molecular weight, as determined by HPLC analysis, was 1,645 Da, and its isoelectric point (pI) was 12.59, indicating a highly cationic character.

In addition to DD-S052P (HLB Science), 11 conventional antimicrobial agents were included in broth microdilution tests to determine minimal inhibitory concentrations (MICs): meropenem (Yuhan Co., Seoul, Korea), ampicillin/sulbactam (Samjin Co., Seoul, Korea), ceftolozane/tazobactam (Pfizer, Madison, NJ, United States; imported by: Korea MSD, Seoul, Korea), colistin (SteriMax Inc., ON, Canada), rifampicin (CKD, Seoul, Korea), minocycline (Sigma-Aldrich, St. Louis, MO, United States), polymyxin B (Sigma-Aldrich, St Louis, MO, United States), tigecycline (Pfizer, Seoul, Korea), levofloxacin (Ildong Pharmaceutical Co., Ltd., Seoul, Korea), ceftazidime/avibactam (Pfizer, Seoul, Korea); and piperacillin/tazobactam (CKD, Seoul, Korea).

### 2.2 Bacterial strains and antimicrobial susceptibility tests

Clinical isolates of CRAB were collected anonymously from the blood cultures of adult patients (≥18 years of age) diagnosed with CRAB bacteremia at Korea University Anam Hospital (Seoul, Korea; IRB registration number: 2022AN0292; 20 June 2022), with a waiver of informed consent. Non-repetitive CRAB isolates were initially identified in a clinical microbiology laboratory using the MicroScan Pos Combo Panel Type 6 automated system (Baxter Diagnostics, West Sacramento, CA, United States) as part of routine diagnostic procedures. Strain identification was subsequently confirmed using matrix-assisted laser desorption/ionization time-of-flight mass spectrometry (Bruker Daltonics, Bremen, Germany).

Antibiotic susceptibility testing of 12 antimicrobial agents was performed using the broth microdilution method to determine their MICs. Each experiment was conducted in triplicate. Testing and interpretation of antibiotic susceptibility breakpoints were performed in accordance with the 2017 Clinical and Laboratory Standards Institute (CLSI) guidelines (Clinical and Laboratory Standards Institute, 2017). Carbapenem resistance was defined as an imipenem MIC of ≥8 mg/L (Clinical and Laboratory Standards Institute, 2017). Two reference bacterial strains, namely, *E. coli* ATCC® 25922 and *Pseudomonas aeruginosa* ATCC® 27853, were used as quality controls for antimicrobial susceptibility testing. Multiplex real-time polymerase chain reaction assays were performed to identify antimicrobial resistance genes in the CRAB isolates (Park et al., 2023).

### 2.3 Synergistic antimicrobial activity of DD-S052P combined with conventional antibiotics

A checkerboard assay was used to evaluate the synergistic activities of two antimicrobial agents. Fifteen CRAB clinical isolates were tested using 96-well plates (Cat. No. 30096; SPL Life Sciences, Pocheon, Korea). The panels of 96-well flat-bottom plates were prepared based on the MIC of each antimicrobial agent, as determined by the broth microdilution method. Dilution intervals were set at 2–8 times and 1/8–1/64 of the MIC values determined in the preliminary analysis. Antibiotic solutions were prepared in cation-adjusted Mueller–Hinton II broth (CA-MHB), with antibiotic-free wells in the lower-left corner of the 96-well plates. After preparing two-fold serial dilutions of both antimicrobial agents, 50 µL of the first antibiotic was dispensed into each row of the plate, and 50 µL of the second antibiotic was added to each column. Consequently, each well contained a 100 µL mixture of the two antimicrobial agents, with varying concentrations across the rows and columns.

The broth-cultured CRAB inoculum in CA-MHB was prepared at twice the desired concentration, based on the 0.5 McFarland standard (100 µL). The final volume in each well was 200 μL, yielding an inoculum concentration of 5 × 105 colony-forming units (CFU)/mL, except in the sterility control well. All plates were then incubated at 37 °C overnight in ambient air. Strains were initially cultured on blood agar plates at 37 °C overnight, followed by a secondary culture in CA-MHB for 2–6 h before use, according to the CLSI guidelines. Bacterial growth was measured using a microplate reader (SpectraMax Plus 384, Molecular Devices, United States) at an optical density of 600 nm (OD600). All assays were performed in triplicate. The fractional inhibitory concentration index (FICI) was calculated as previously described (Ju et al., 2022) and interpreted as follows: FICI ≤ 0.5, synergistic; 0.5 < FICI ≤ 1, additive; 1 < FICI ≤ 2, indifferent; and FICI > 2, antagonistic (Ju et al., 2022). For each well, the reported FICI corresponded to the value observed in at least two of three replicate experiments, and there were no instances in which all three values differed.

### 2.4 Bacterial growth inhibition and time-killing assay

First, to screen for synergistic effects of various antibiotic combinations, a time-kill assay was performed using a randomly selected CRAB isolate. Subsequently, time-kill assays were conducted for the three combinations expected to produce reproducible synergistic effects across the remaining 14 CRAB isolates. In brief, tubes containing freshly prepared CA-MHB supplemented with antimicrobial agents, alone or in combination, were inoculated with CRAB isolates at a concentration of 5 × 10^5^ CFU/mL. The inoculum was prepared using the McFarland 0.5 standard (Schofield, 2012). Each tube containing 30 mL of suspension was incubated at 37 °C with shaking at 200 rpm under ambient conditions. Next, 100 μL aliquots were collected from each tube at specified time points (0, 2, 4, 8, 12, and 24 h of incubation) and serially diluted in saline to measure bacterial growth. Following a 1:10 serial dilution, 100 µL was plated onto blood agar for colony counting and incubated overnight at 37 °C. Colony counts were used to estimate the initial bacterial density of the original sample based on the dilution factor. The lower limit of detection was 1 log_10_ CFU/mL, as defined by the detection capability of the method. The antibiotic concentrations for monotherapy were set at 1 × MIC for each antimicrobial agent. For combination therapy, concentrations of 0.5 × MIC, 1 × MIC, and 2 × MIC were used, as determined based on the individual MIC values of each antibiotic in the combination. For example, the 0.5 × MIC condition for the DD-S052P + SAM combination consisted of 0.5 × MIC of DD-S052P with 0.5 × MIC of SAM. The antibiotic combinations tested were identical to those used in the checkerboard assay.

For both single antimicrobial agents and their combinations, bactericidal activity was defined as ≥ 3 log_10_ reduction in viable cell count within 24 h compared with that at the initial time point. A synergistic effect was defined as a ≥2 log_10_ CFU/mL reduction within 24 h for the combination compared with the most active individual agent at different time points (Wences et al., 2022). An increase of >2 log_10_ was considered indicative of antagonism (Pankey et al., 2014). Indifference was defined as any outcome that did not meet the criteria for synergy or antagonism (Pankey et al., 2014).

### 2.5 Mouse infection model

Immunocompetent specific pathogen-free CD-1 (Institute of Cancer Research; ICR) young male mice (average weight 35 ± 3 g; 8 weeks old) were supplied by Orient Bio Inc. (Seongnam, Korea). Prior to experimentation, mice were housed under specific-pathogen-free conditions for 1 week to allow acclimatization. All mice were maintained in the infectious animal facility of Korea University, which operates under ABSL-2 conditions.

A CRAB isolate was inoculated into MHB and incubated for 6 h, followed by cell harvesting via centrifugation. The supernatant was discarded, and the bacterial pellet was resuspended in saline to adjust the concentration to 2 × 10^8^ CFU/mL using a McFarland densitometer (DEN-1B) (Harris et al., 2013). For the CRAB challenge, a 500 µL suspension (yielding approximately 20% survival) was injected into the central abdomen of healthy mice using an insulin syringe at a 90° angle to a depth of approximately 4 mm. To evaluate the efficacy of each antimicrobial agent as monotherapy, the agents were administered 30 min after bacterial inoculation to allow for bacterial proliferation and spread (Kleinhenz et al., 2022). Assuming a mouse body weight of 40 g, seven antimicrobial agents were prepared at five concentrations each using two-fold serial dilutions. Each antibiotic solution was dissolved in saline, and a 200 µL solution was prepared for administration. Drugs were administered in 50 µL injections at four separate sites (Hoyle et al., 2020). Two concentrations of each drug were used: the lowest concentration (H) that achieved the highest survival rate as a single dose and one-quarter of that concentration (L). The same combinations tested in the checkerboard assays were used, with antibiotics prepared at twice the concentrations employed under single-agent conditions. A 100 µL injection of each drug, prepared at twice the final concentration, was administered, resulting in a total injection volume of 200 µL. In brief, combinations of drugs A and B—A(H)–B(H), A(H)–B(L), A(L)–B(H), and A(L)–B(L)—were prepared. The A(H)-B(H) combination was administered as four 50 µL injections (A at 2 × H, B at 2 × H, A at 2 × H, and B at 2 × H) at 30 min intervals, for a total of 200 µL. Saline was administered as a control following the same injection schedule. Following bacterial inoculation into the abdominal umbilical region of the mice, antibiotics were administered to all four abdominal quadrants: upper right, lower right, upper left, and lower left. The antibiotic injection sites were located approximately 1.5 cm from the bacterial injection site. The study endpoint was set at 48 h. Each experimental group included five mice, and each antibiotic combination set included five experimental groups: control, H–H, H–L, L–H, and L–L. A total of 15 antibiotic combination sets were tested, resulting in 75 experimental groups; the experiment was performed once. All animal experiments were approved by the Institutional Animal Care and Use Committee of Korea University (Approval No. KOREA-2025-0035) and conducted in accordance with the National Institutes of Health Guide for the Care and Use of Laboratory Animals. During the experiments, mice were maintained under standard laboratory conditions with *ad libitum* access to food and water.

## 3 Results

### 3.1 Characteristics of *A. baumannii* clinical isolates

The clinical and microbiological characteristics of the CRAB isolates included in this study are presented in Table 1. All patients from whom CRAB isolates were isolated from blood samples succumbed to CRAB infection. All 15 clinical isolates were resistant to meropenem, with an MIC of ≥16 mg/L. All isolates carried the OXA-51-type and OXA-23-type carbapenem-hydrolyzing oxacillinases but did not harbor class B metallo-carbapenemases or other class D carbapenemases. The susceptibility rates, MIC_50_, MIC_90_, and MIC range associated with each conventional antibiotic are summarized in Supplementary Table S1.

**TABLE 1:** Clinical and microbiological characteristics of *Acinetobacter baumannii* isolates.

Isolate no	Patient information				MIC of antimicrobial agents (mg/L)^a^											
	Age	Sex	Clinical specimen	In-hospital mortality	MEM	SAM	C/T	CST	RIF	MIN	PMB	TGC	LVX	CZA	TZP	DD-S052P
ATCC 25922	-	-	-	-	-	4	<0.25	2	8	0.25	0.25	<0.25	<0.25	<0.25	0.5	4
ATCC 27853	-	-	-	-	0.5	-	0.5	2	16	-	-	-	1	1	2	64
1	71	F	Blood	Yes	**16**	**16**	1	**16**	4	<0.125	0.5	0.5	1	2	**64**	16
2	65	F	Blood	Yes	**64**	**64**	16	**8**	16	0.25	0.5	2	**16**	32	**>256**	64
3	67	F	Blood	Yes	**64**	**32**	16	**8**	2	0.25	0.5	2	**16**	32	**>256**	64
4	75	M	Blood	Yes	**64**	**64**	32	**8**	2	0.25	0.5	2	**32**	32	**256**	64
5	84	F	Blood	Yes	**64**	**64**	32	2	2	4	0.25	2	**256**	32	**256**	64
6	61	M	Blood	Yes	**32**	**64**	32	2	2	4	0.25	2	**16**	32	**256**	64
7	63	M	Blood	Yes	**64**	**32**	16	**8**	4	0.5	0.5	2	**64**	32	**256**	16
8	90	F	Blood	Yes	**32**	**32**	64	**8**	2	0.25	0.5	2	2	32	**256**	32
9	73	M	Blood	Yes	**64**	**64**	8	**16**	4	0.5	0.5	2	**64**	32	**>256**	16
10	92	F	Blood	Yes	**64**	**64**	32	**4**	2	4	0.25	1	**8**	16	**>256**	32
11	63	M	Blood	Yes	**32**	**64**	16	**16**	4	0.5	0.5	0.5	**32**	16	**256**	16
12	90	M	Blood	Yes	**16**	**16**	64	**4**	2	0.5	0.25	1	**4**	32	**256**	32
13	65	F	Blood	Yes	**64**	**16**	128	**4**	2	0.5	0.25	1	**4**	32	**>256**	32
14	45	M	Blood	Yes	**32**	**128**	128	**16**	8	1	0.5	0.5	**4**	32	**256**	32
15	54	M	Blood	Yes	**32**	**32**	16	**16**	2	0.25	0.5	1	**4**	16	**256**	64

^a^ Bold values, non-susceptible (Clinical and Laboratory Standards Institute, 2017). C/T, ceftolozane/tazobactam; CST, colistin; CZA, ceftazidime/avibactam; F, female; LVX, levofloxacin; M, male; MEM, meropenem; MIC, minimum inhibitory concentration; MIN, minocycline; PMB, polymyxin B; RIF, rifampicin; SAM, ampicillin/sulbactam; TGC, tigecycline; TZP, piperacillin/tazobactam.

### 3.2 Checkerboard assay against *A. baumannii* clinical isolates

Checkerboard assays performed against the 15 CRAB isolates revealed that DD-S052P exhibited *in vitro* synergistic effects when combined with select conventional antibiotics (Table 2). Synergistic effects were confirmed when observed in at least two of three replicate experiments. Among DD-S052P-based combinations, DD-S052P plus meropenem exhibited the highest *in vitro* synergistic activity against CRAB isolates (100%), followed by DD-S052P plus minocycline (93%), DD-S052P plus ampicillin/sulbactam (93%), DD-S052P plus ceftolozane/tazobactam (80%), and DD-S052P plus colistin (80%). In contrast, DD-S052P plus tigecycline showed a considerably lower synergistic rate of 13% (Table 2). Overall, in the checkerboard assay, combination therapies excluding DD-S052P exhibited lower synergistic activity than DD-S052P-based combinations. Notably, tigecycline-based combinations did not demonstrate significant synergistic effects, whereas the colistin-tigecycline combination showed antagonism in 80% of the 15 CRAB isolates (Table 2). The representative raw OD_600_ values from the checkerboard assay are provided in Supplementary Table S2. The FICI values calculated from these data are summarized in Supplementary Table S3.

**TABLE 2:** Results of checkerboard assay for two-drug combinations against clinical isolates of *Acinetobacter baumannii*. Gray cells indicate a synergistic effect.

Isolate	DDSP + MIN	DDSP + SAM	DDSP + MEM	DDSP + C/T	DDSP + TGC	DDSP + CST	CST + MIN	CST+ SAM	CST + C/T	CST + MEM	CST + TGC	TGC + MEM	TGC + C/T	TGC + SAM	TGC + MIN
1	1.500	0.516	0.500	0.750	0.750	0.500	0.500	0.504	1.000	1.004	0.501	0.750	1.125	0.516	0.500
2	0.250	0.250	0.070	0.266	0.625	0.281	1.031	1.008	1.004	0.531	2.500	1.031	0.625	0.516	0.625
3	0.313	0.258	0.188	0.313	1.000	0.500	1.000	0.531	1.000	0.625	2.500	0.750	1.016	0.563	0.625
4	0.156	0.375	0.047	0.250	0.531	0.313	0.625	0.504	0.563	0.750	2.250	0.625	1.000	1.000	1.000
5	0.281	0.375	0.125	0.250	0.313	0.313	0.500	0.625	0.250	0.375	2.500	0.750	0.625	0.375	0.281
6	0.281	0.313	0.188	0.313	0.750	0.375	1.000	0.625	0.500	0.375	2.500	1.000	0.508	0.563	1.125
7	0.375	0.375	0.375	0.500	0.625	0.563	0.625	0.504	0.750	0.563	4.250	1.000	0.750	1.000	0.563
8	0.250	0.375	0.250	0.500	0.563	0.313	0.750	0.750	0.563	0.516	4.125	1.063	0.750	0.750	0.750
9	0.375	0.375	0.375	0.531	1.063	0.625	0.750	1.000	1.004	0.504	2.250	1.063	0.750	0.750	0.750
10	0.281	0.375	0.133	0.250	0.188	0.313	2.125	0.563	1.000	0.750	1.004	0.563	2.008	0.563	0.750
11	0.250	0.500	0.063	0.531	0.750	0.750	0.625	0.750	1.004	0.750	2.250	1.000	1.000	1.000	0.625
12	0.375	0.375	0.375	0.313	0.625	0.250	1.125	0.500	0.750	0.625	4.125	0.750	1.000	0.750	0.563
13	0.375	0.500	0.188	0.375	0.563	0.375	0.750	0.500	0.625	0.750	2.500	0.750	0.750	1.000	0.750
14	0.188	0.258	0.141	0.252	0.625	0.188	0.625	0.750	1.008	0.531	1.002	0.750	1.000	1.002	0.750
15	0.313	0.250	0.250	0.188	0.625	0.281	0.625	0.750	0.625	1.000	2.250	1.000	1.000	2.063	1.000

%	DDSP + MIN	DDSP + SAM	DDSP + MEM	DDSP + C/T	DDSP + TGC	DDSP + CST	CST + MIN	CST + SAM	CST + C/T	CST + MEM	CST + TGC	TGC + MEM	TGC + C/T	TGC + SAM	TGC + MIN
Syn	93.33	93.33	100.00	80.00	13.33	80.00	13.33	13.33	13.33	13.33	0.00	0.00	0.00	6.67	13.33
Add	0.00	6.67	0.00	20.00	80.00	20.00	66.67	80.00	60.00	80.00	6.67	80.00	80.00	80.00	80.00
Ind	6.67	0.00	0.00	0.00	6.67	0.00	13.33	6.67	26.67	6.67	13.33	20.00	13.33	6.67	6.67
Anta	0.00	0.00	0.00	0.00	0.00	0.00	6.67	0.00	0.00	0.00	80.00	0.00	6.67	6.67	0.00

The fractional inhibitory concentration index (FICI) was interpreted as follows: FICI ≤ 0.5, synergistic (dark gray); 0.5 < FICI ≤ 1, additive (medium gray); 1 < FICI ≤ 2, indifferent (light gray); and FICI > 2, antagonistic (white) (Ju et al., 2022).

Add, additive; Anta, antagonism; C/T, ceftolozane/tazobactam; CST, colistin; DDSP, DD-S052P; Ind, indifferent; MEM, meropenem; MIN, minocycline; SAM, ampicillin/sulbactam; Syn, synergistic; TGC, tigecycline.

### 3.3 Time-kill assay against *A. baumannii* clinical isolates

To evaluate the time-dependent effects of the antibiotic combinations, a time-kill assay was performed on a randomly selected CRAB isolate (No. 3) using the same combination regimens tested in the checkerboard assay (Supplementary Table S4).

The bactericidal effects of antibiotic monotherapy (1 × MIC) and combination therapy (0.5 × MIC, 1 × MIC, and 2 × MIC) against the CRAB isolate were evaluated. Meropenem monotherapy exhibited bactericidal activity, whereas ceftolozane/tazobactam, tigecycline, and minocycline showed bacteriostatic effects. Notably, monotherapy with ampicillin/sulbactam, colistin, and DD-S052P did not exhibit the expected antibacterial activity at 24 h (1 × MIC; Supplementary Table S5). A ceiling effect was observed with meropenem monotherapy, precluding the assessment of its synergistic effects when combined with conventional antibiotics. No antagonism was observed in the time-kill assay. At an antibiotic concentration of 0.5 × MIC, DD-S052P combined with ampicillin/sulbactam, ceftolozane/tazobactam, tigecycline, and colistin exhibited both *in vitro* synergistic and bactericidal effects against the CRAB isolate at 24 h in the time-kill assay (Table 3). Among combination therapies excluding DD-S052P, colistin plus minocycline, colistin plus tigecycline, and tigecycline plus ceftolozane/tazobactam demonstrated both bactericidal and synergistic effects at 24 h (Table 3).

**TABLE 3:** Time-kill assay results showing the effects of antibiotic combinations against strain No. 3 at different concentrations at 24 h.

Drug combinations	24 h MIC		
	0.5 × MIC	1 × MIC	2 × MIC
DDSP + SAM	B	B	B
DDSP + C/T	B	B	B
DDSP + MIN		B	B
DDSP + MEM^a^	B	B	B
DDSP + TGC	B	B	B
DDSP + CST	B	B	B
CST + SAM		B	B
CST + C/T		B	B
CST + MIN	B	B	B
CST + MEM^a^		B	B
CST + TGC	B	B	B
TGC + SAM		B	B
TGC + C/T	B	B	B
TGC + MIN		B	B
TGC + MEM^a^		B	B

Gray shading indicates synergy. B indicates bactericidal activity (≥3 log_10_ CFU reduction within 24 h).

^a^ At 1 × MIC, meropenem monotherapy completely inhibits CRAB, preventing the assessment of its combinatorial effects with other conventional antibiotics.

C/T, ceftolozane/tazobactam; CST, colistin; DDSP, DD-S052P; MEM, meropenem; MIC, minimum inhibitory concentration; MIN, minocycline; SAM, ampicillin/sulbactam; TGC, tigecycline.

When combined with DD-S052P, ampicillin/sulbactam, ceftolozane/tazobactam, and colistin exhibited *in vitro* synergistic effects at 24 h in both the checkerboard and time-kill assays (0.5 × MIC). Furthermore, all combinations exhibited bactericidal effects against the CRAB isolates in the time-kill assay. When combined with DD-S052P, ampicillin/sulbactam, ceftolozane/tazobactam, tigecycline, and colistin also exhibited synergistic and bactericidal effects at all tested concentrations (0.5 × MIC, 1 × MIC, and 2 × MIC). Notably, DD-S052P combined with colistin, ampicillin/sulbactam, or tigecycline demonstrated bactericidal activity at all examined time points (Supplementary Figure S2). DD-S052P combined with colistin achieved bacterial killing at a lower colistin concentration (4 mg/L) than colistin alone (8 mg/L). At a minimal media concentration of 0.5 × MIC, the combination was bactericidal, with bacterial killing occurring up to 2 h faster than with colistin alone.

When combined with DD-S052P, ampicillin/sulbactam, ceftolozane/tazobactam, and colistin demonstrated synergistic effects against all 15 CRAB strains (1.0 × MIC). Excluding strains exhibiting a ceiling effect, bactericidal activity was observed in 86.7%, 86.7%, and 100% of isolates for DD-S052P combined with ampicillin/sulbactam, ceftolozane/tazobactam, and colistin, respectively (Table 4).

**TABLE 4:** Results of time-kill assay and bactericidal activity of two-drug combinations (1 × MIC at 24 h) against 15 clinical isolates of *Acinetobacter baumannii*.

Isolate	DDSP+SAM	DDSP+C/T	DDSP+CST
1	B	B	
2	B	B	
3	B	B	B
4	B	B	B
5	B	B	B
6	B	B	B
7	B	B	B
8	B	B	B
9	B		
10		B	
11			
12	B	B	B
13	B	B	B
14	B	B	
15	B	B	

Gray shading indicates synergy. B indicates bactericidal activity (≥3 log_10_ CFU reduction within 24 h). The white cell indicates a ceiling effect, precluding confirmation of either synergy or bactericidal activity.

C/T, ceftolozane/tazobactam; CST, colistin; DDSP, DD-S052P; SAM, ampicillin/sulbactam.

### 3.4 Effects on survival of the mouse infection model

**FIGURE 1:**
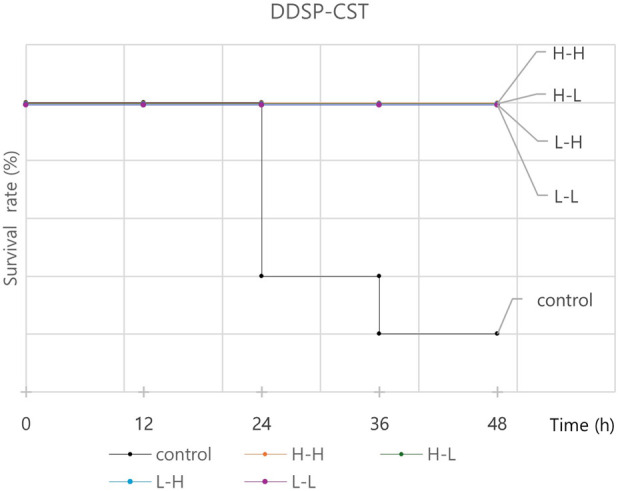
Kaplan–Meier survival curve of mice infected with carbapenem-resistant *Acinetobacter baumannii* (CRAB) and treated with the DD-S052P and colistin combination. High-dose DD-S052P (10 mg/kg), low-dose DD-S052P (2.5 mg/kg), high-dose colistin (10 mg/kg), and low-dose colistin (2.5 mg/kg). CST, colistin; DDSP, DD-S052P; H, high dose; L, low dose.

Table 5 summarizes the 48 h mortality rates in mouse models treated with various regimens. Antibiotic concentrations used in the animal experiments are provided in Supplementary Table S6. In CRAB-infected mice, low-dose administration of two-drug combinations resulted in 100% survival for the following combinations: DD-S052P plus minocycline, DD-S052P plus colistin, colistin plus ceftolozane/tazobactam, colistin plus minocycline, and tigecycline plus minocycline. Notably, the DD-S052P plus colistin combination was the only regimen that consistently demonstrated synergistic effects across the checkerboard, time-kill, and animal experiments. The Kaplan–Meier survival curve for the DD-S052P plus colistin combination is shown in Figure 1 as a representative curve. Survival curves for other experimental groups are presented in Supplementary Figure S3.

**TABLE 5:** Survival rates at 48 h in antimicrobial-treated mouse models.

48 h survival	Monotherapy	Combination therapy																			
	Control	H	L	DDSP-based combination	Control	H–H	H–L	L–H	L–L	CST-based combination	Control	H–H	H–L	L–H	L–L	TGC-based combination	Control	H–H	H–L	L–H	L–L
MEM	0%	60%	20%	MEM	20%	100%	60%	80%	40%	MEM	0%	100%	100%	80%	40%	MEM	20%	100%	80%	100%	80%
C/T	0%	60%	20%	C/T	20%	60%	40%	20%	0%	C/T	40%	100%	100%	100%	100%	C/T	0%	80%	100%	60%	80%
SAM	0%	40%	20%	SAM	40%	0%	40%	20%	0%	SAM	20%	100%	100%	20%	40%	SAM	0%	80%	80%	40%	60%
MIN	H 0% L 40%	100%	60%	MIN	20%	60%	40%	80%	100%	MIN	40%	100%	100%	100%	100%	MIN	40%	100%	100%	100%	100%
TGC	20%	100%	20%	TGC	20%	100%	80%	80%	80%	TGC	20%	100%	100%	100%	80%	-	​	​	​	​	​
CST	40%	100%	40%	CST	20%	100%	100%	100%	100%	-	​	​	​	​	​	CST	20%	100%	100%	100%	80%
DDSP	20%	20%	20%	-	​	​	​	​	​	DDSP	20%	100%	100%	100%	100%	DDSP	20%	100%	80%	80%	80%

For detailed information on high and low doses for each antibiotic, see Supplementary Table S6.

C/T, ceftolozane/tazobactam; CST, colistin; DDSP, DD-S052P; H, the lowest dose that achieved the highest survival rate; L, one-quarter of H dose; MEM, meropenem; MIN, minocycline; SAM, ampicillin/sulbactam; TGC, tigecycline.

## 4 Discussion

Our findings suggest that DD-S052P has potential as an adjuvant when combined with conventional antibiotics, particularly colistin, for the treatment of CRAB infections. However, its efficacy as monotherapy remains unconfirmed, and its potential as a promising adjuvant in animal models warrants further validation.

The mechanism by which DD-S052P acts as an adjuvant in combination with colistin remains unclear. We confirmed that DD-S052P strongly binds to LPS, a major component of the outer membrane of Gram-negative bacteria, thereby disrupting the bacterial cell membrane and contributing to its antibacterial activity (Supplementary Figures S1B, S1C). Similar to DD-S052P, well-known antimicrobial peptides such as papiliocin and melittin reportedly adopt amphipathic cationic α-helical structures that bind to negatively charged LPS and disrupt bacterial membrane integrity through electrostatic and hydrophobic interactions (Kim et al., 2011; Wang, 2008). However, the precise mechanism of action of DD-S052P has not yet been clearly elucidated.

Antimicrobial peptides can be classified into four structural groups: linear α-helical peptides, β-sheet peptides, linear random-coil structures, and peptides containing both α-helix and β-sheet configurations (Pushpanathan et al., 2013; Lei et al., 2019). DD-S052P is an 11-amino acid peptide that adopts an α-helical structure with amphipathic properties, facilitating strong interactions with LPS (Supplementary Figures S1B, S1D). Accumulation of DD-S052P on the bacterial cell membrane surface, or its aggregation through vertically embedding into the bilayer of the cell membrane, leads to leakage of cellular contents and, ultimately, cell death (Supplementary Figure S1C) (Lei et al., 2019; Huan et al., 2020).

In addition to their immediate antimicrobial actions, antimicrobial peptides may exhibit anti-inflammatory, antiendotoxin, regenerative (skin and bone), and anticancer properties (Taute et al., 2019; Liang and Diana, 2020; de Souza et al., 2022; Li et al., 2023; Li et al., 2025; Zhu et al., 2025). In our experiments, the combination of DD-S052P and minocycline, which did not demonstrate synergistic or bactericidal effects in *vitro* time-kill assays (0.5 × MIC), achieved excellent survival rates *in vivo* at low combination levels. Although the influence of antimicrobial agent concentrations used in the mouse model cannot be completely ruled out, the beneficial multifaceted effects of DD-S052P on survival, such as immunomodulation and intracellular targeting, need to be clarified through additional experiments. Nevertheless, improved treatment outcomes were not observed with all conventional antibiotic combinations.

Combination therapy using two or more antimicrobial agents is a promising strategy for combating MDR bacteria. The use of antimicrobial peptides in combination with conventional antibiotics can enhance overall treatment efficacy, help overcome resistance, and increase inhibitory activity against MDR bacteria. In particular, their synergistic effect can reduce the required doses of conventional antibiotics, thereby lowering the risk of drug toxicity and selection pressure associated with high antibiotic use (Yang et al., 2024).

In this study, the combination of DD-S052P and colistin emerged as the most promising treatment option for CRAB infections. This regimen controlled CRAB more rapidly and at lower concentrations than colistin alone. Colistin is widely regarded as a last-resort antibiotic for treating infections caused by MDR Gram-negative bacteria. However, robust evidence supporting colistin efficacy against CRAB infections is limited due to its considerable nephrotoxicity and neurotoxicity, along with the emergence of resistance in clinical settings (Lim et al., 2010). Recent studies have suggested synergistic effects when antimicrobial peptides are combined with conventional antibiotics against CRAB, specifically tridecaptin M, polycyclic peptide nisin, and esculentin 1-21 (Jangra et al., 2020; Jahangiri et al., 2021; Witherell et al., 2021; Sacco et al., 2022). Notably, both esculentin 1-21 and colistin demonstrated markedly enhanced efficacy when administered at sub-inhibitory concentrations (Sacco et al., 2022). Reducing the colistin dose is expected to improve therapeutic outcomes by mitigating toxicity and consequently reducing the emergence of resistance.

First, colistin may penetrate cell membranes more quickly due to the higher pI of DD-S052P. Colistin exerts its antibacterial effect by displacing divalent cations, such as calcium or magnesium, which stabilize the LPS structure, a key component of the outer membrane of Gram-negative bacteria (Khadka et al., 2018). Colistin has a pI value of 10.4, whereas DD-S052P has a pI value of 12.59, which is considerably higher than the pI value of 9.2 of other antimicrobial peptides, indicating a strong positive charge (Lewis and Lewis, 2004; Torrent et al., 2011; Clifton et al., 2015; Mandal et al., 2024). Consequently, DD-S052P may facilitate outer-membrane permeabilization, thereby accelerating colistin access to its intracellular targets and improving its antibacterial efficacy against CRAB when used in combination.

Second, DD-S052P, with a strong positive charge, may neutralize the negative charge of the methanesulfonate groups, thereby promoting the rapid conversion of colistin methanesulfonate (CMS), the prodrug, into colistin, the active drug. Clinically, colistin is administered intravenously as CMS, which lacks intrinsic antibacterial activity, is less toxic, and is hydrolyzed *in vivo* to yield colistin (Bergen et al., 2006). Rapid conversion of CMS to active colistin allows the target concentration to be reached more quickly at a lower dose, thereby reducing medical costs and the risk of adverse events. Notably, the rapid attainment of therapeutic colistin concentrations in the blood could markedly improve treatment outcomes in patients with severe infections, such as septic shock (Guo et al., 2025).

In our study, the colistin plus tigecycline combination exhibited a high antagonism rate of 80%. A previous study suggested that the strong bacteriostatic activity of tigecycline inhibits bacterial metabolism, which may, in turn, reduce the efficacy of colistin (Cikman et al., 2015). Similarly, the DD-S052P plus tigecycline combination showed predominantly additive effects (80%) in the checkerboard assay, whereas DD-S052P-based combinations with other conventional antibiotics demonstrated high synergistic rates. These findings suggest a potential shared mechanism of action between colistin and DD-S052P targeting LPS in bacterial cell membranes.

Our study has some limitations. First, discrepancies between *in vitro* and *in vivo* experiments may arise from various uncharacterized factors present under *in vivo* conditions. For example, minocycline and tigecycline exhibited poor efficacy *in vitro* but favorable efficacy *in vivo*. Tetracyclines are known to possess anti-inflammatory properties, which may enhance their *in vivo* efficacy through interactions with the host immune system. In addition, the prodrug CMS is converted to the active form, colistin, *in vivo* and may exhibit enhanced antibacterial activity compared with that observed *in vitro* (Bergen et al., 2006). Second, we used a mouse model of peritoneal infection, and the antibacterial activity of each antibiotic may not be directly generalizable to other animal models. In particular, the enhanced efficacy of tigecycline may be attributed to its lipophilicity, which promotes accumulation within the peritoneal cavity (Rubino et al., 2007). Third, mouse experiments were performed with only one CRAB strain. Consequently, we were unable to assess the efficacy of DD-S052 against diverse resistance mechanisms or varying antimicrobial susceptibility. Finally, several antibiotics administered at high concentrations exhibit near-maximal survival when used as monotherapy, resulting in a ceiling effect that limits the ability to observe additional benefits from combination therapy (Andrade, 2021). The ceiling effect occurred when the survival rate of mice reached 100%, preventing the detection of any further improvements in drug efficacy. Therefore, evaluation of synergistic effects is most feasible with combination therapy at low doses, where the ceiling effect is less likely to occur.

Our findings suggest that DD-S052P may serve as an antimicrobial adjuvant for colistin, offering an alternative strategy for treating CRAB infections, likely by potentiating its membrane-disrupting action. Combination therapy with DD-S052P may enhance the efficacy of colistin, which is currently associated with suboptimal treatment success rates. Nevertheless, the clinical application of this combination therapy warrants further investigation, including elucidation of its mechanism of action and validation in clinical studies.

## Data Availability

The original contributions presented in the study are included in the article/Supplementary Material; further inquiries can be directed to the corresponding author.
