# Correction: Peptide-based antimicrobial effect against carbapenem-resistant *Acinetobacter baumannii*: preclinical drug assessment and translational potential

**DOI:** 10.3389/fphar.2026.1817394

**Published:** 2026-03-09

**Authors:** Hak Jun Lee, Seung Jun Lee, Yoon Hyun Sung, Seung Min Park, Seung Pyo Choi, Yangmee Kim, Young Kyung Yoon

**Affiliations:** 1 Institute of Emerging Infectious Diseases, Korea University, Seoul, Republic of Korea; 2 R&D Team, HLB Science, Seoul, Republic of Korea; 3 Department of Bioscience and Biotechnology, Konkuk University, Seoul, Republic of Korea; 4 Division of Infectious Diseases, Department of Internal Medicine, Korea University College of Medicine, Seoul, Republic of Korea

**Keywords:** *Acinetobacter* baumannii, animal model, antimicrobial peptides, carbapenem-resistant, combination therapy

An incorrect number was provided for the grant from the Korea Health Technology R&D Project through the Korea Health Industry Development Institute, funded by the Ministry of Health and Welfare, Republic of Korea. The correct number is **HI23C1297**.

The original article has been updated.

