# Supplementary material for: Peptide-based antimicrobial effect against carbapenem-resistant Acinetobacter baumannii: preclinical drug assessment and translational potential
Source: Front Pharmacol. 2026 Feb 18;17:1732644. doi: 10.3389/fphar.2026.1732644 (PMC12957892; doi:10.3389/fphar.2026.1732644)
Supplement: Supplementary file 1 [file Supplementaryfile1.docx]

Supplementary Material

# Supplementary Figures and Tables

## Supplementary Figures


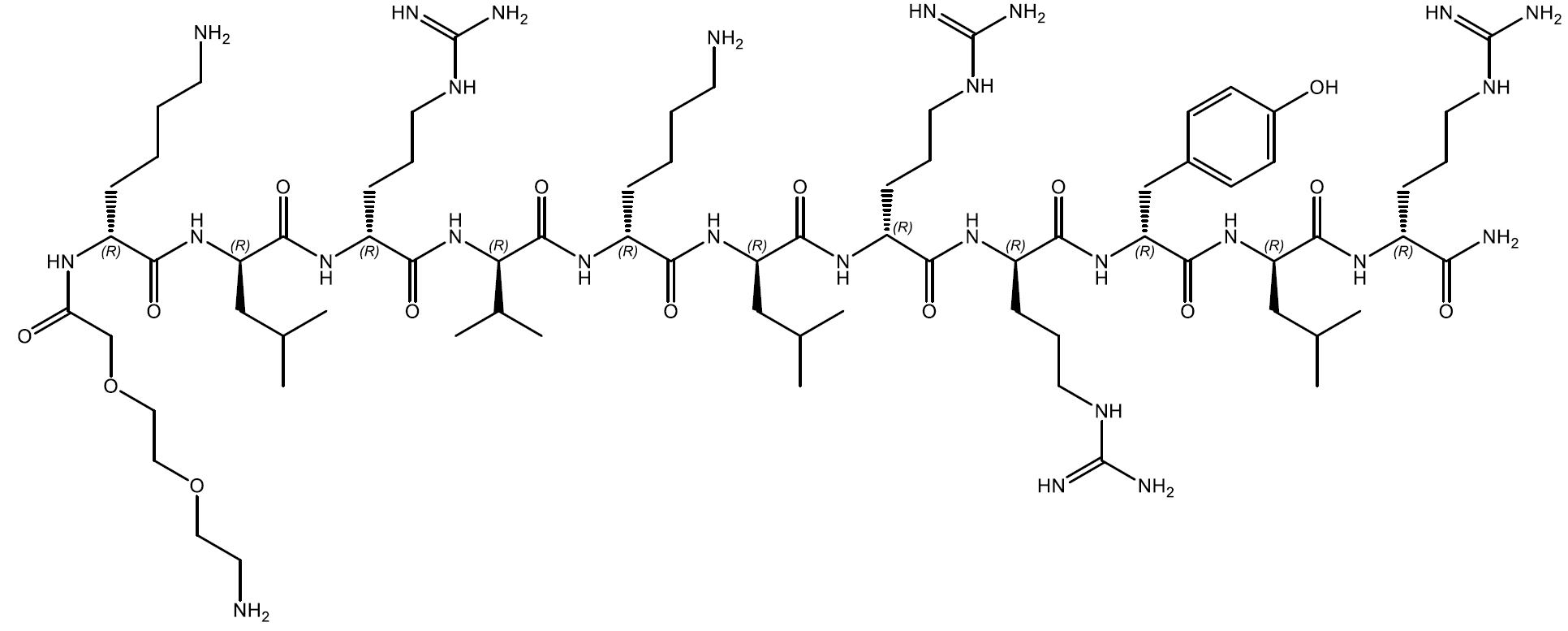


**A**

**B**


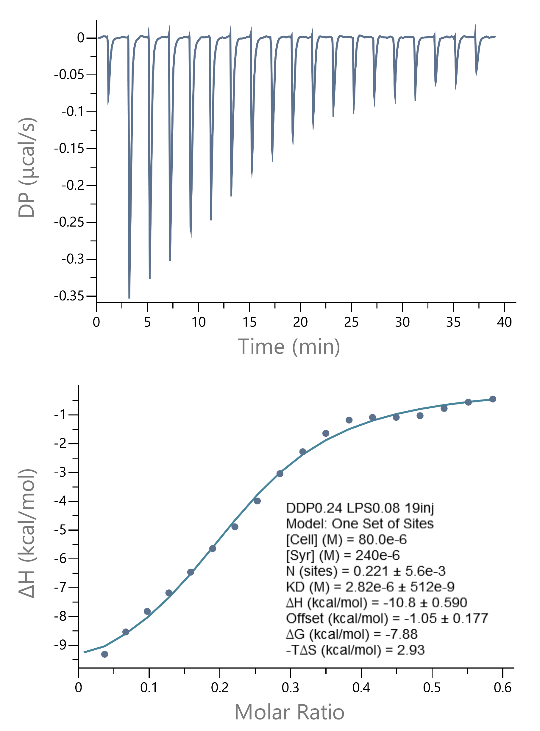

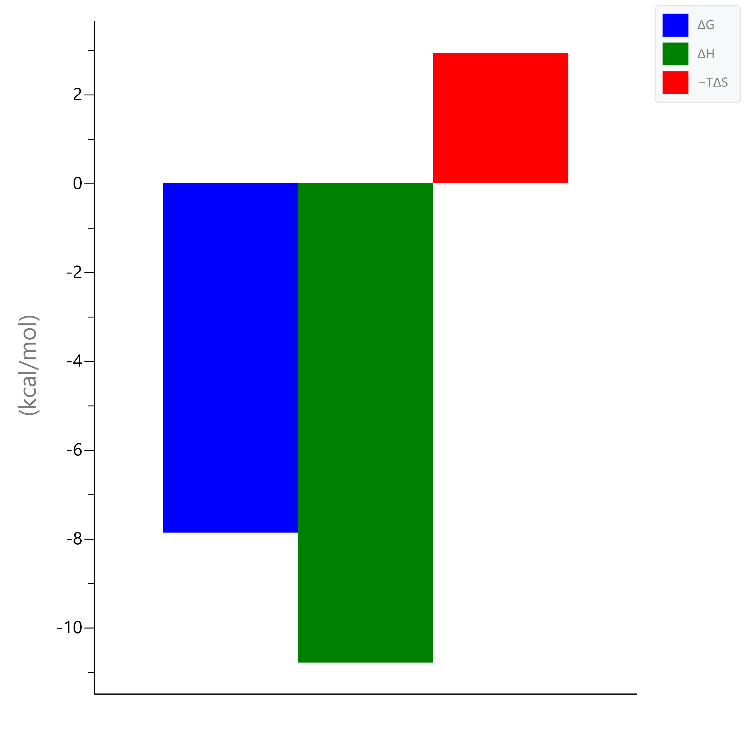


LPS 0.08 mM : DD-S052P 0.24 mM (1:3)


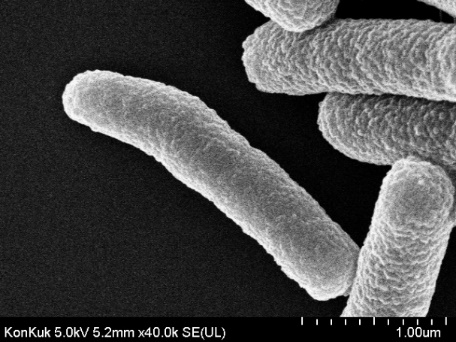

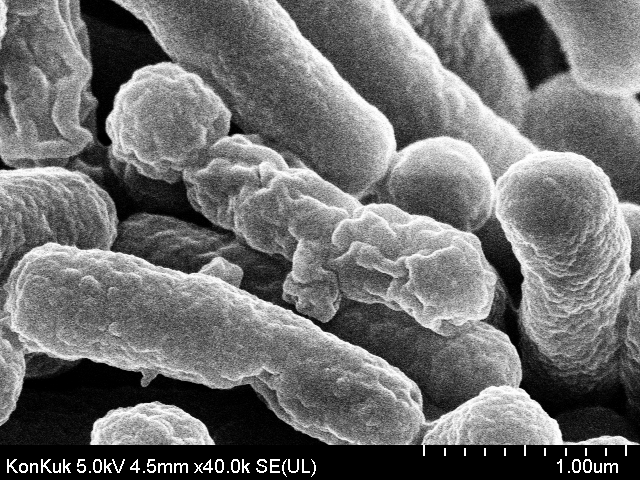

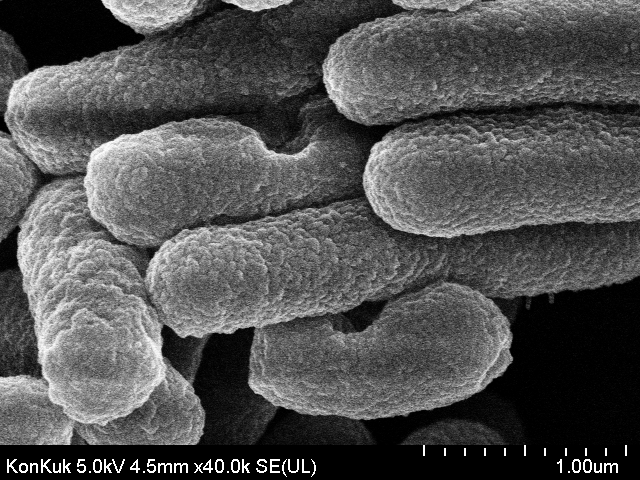

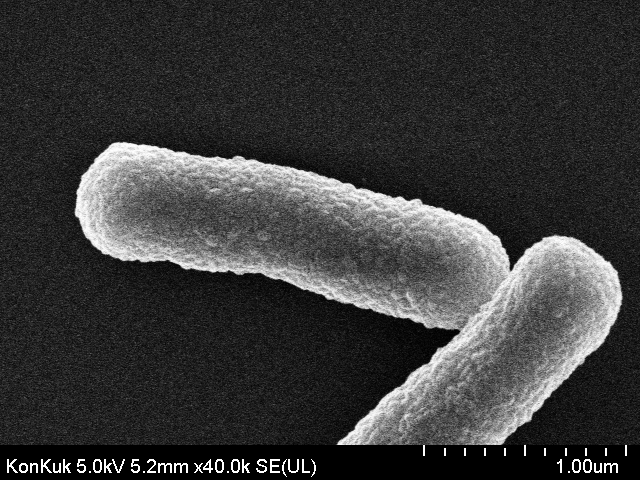


**C**

(a) *E.coli* (control)

(b) DD-S052P/ 1 h

DD-S052P/ 2 h

*E.coli (control)*

DD-S052P

L-form peptide

DD-S052P

L-form peptide

**D**

**Supplementary Figure 1.** Structure and bactericidal effects of DD-S052P. (A) Chemical structure of DD-S052P. DD-S052P is a synthetic linear 11-amino acid peptide with a free amino group (AEEAc) at the N-terminus and carboxamide at the C-terminus. All residues are in D-configuration. (B) Thermodynamic analysis of the lipopolysaccharide (LPS)‒DD-S052P complex. DD-S052P exhibits strong binding affinity to LPS at the micromolar level and demonstrates an exothermic reaction. The dissociation constant (Kd) reflects a strong binding affinity, a negative Gibbs free energy change (ΔG) indicates spontaneous binding, and a negative enthalpy change (ΔH) represents an exothermic reaction. (C) Morphological changes in the cell membrane of *Escherichia coli* treated with 4 μM DD-S052P (scanning electron microscope; scale bar = 1 μm). At 1 h, the cell membrane appears rough. At 2 h, the cell membrane is disrupted with holes, and the cells appear shrunken. (D) Circular dichroism (CD) analysis of DD-S052P. DD-S052P exhibits a random-coil structure in aqueous solution and adopts an α-helical conformation under bacterial membrane–mimicking conditions. The chirality change induced by D-amino acid substitution is confirmed by its spectral difference from the enantiomeric peptide.


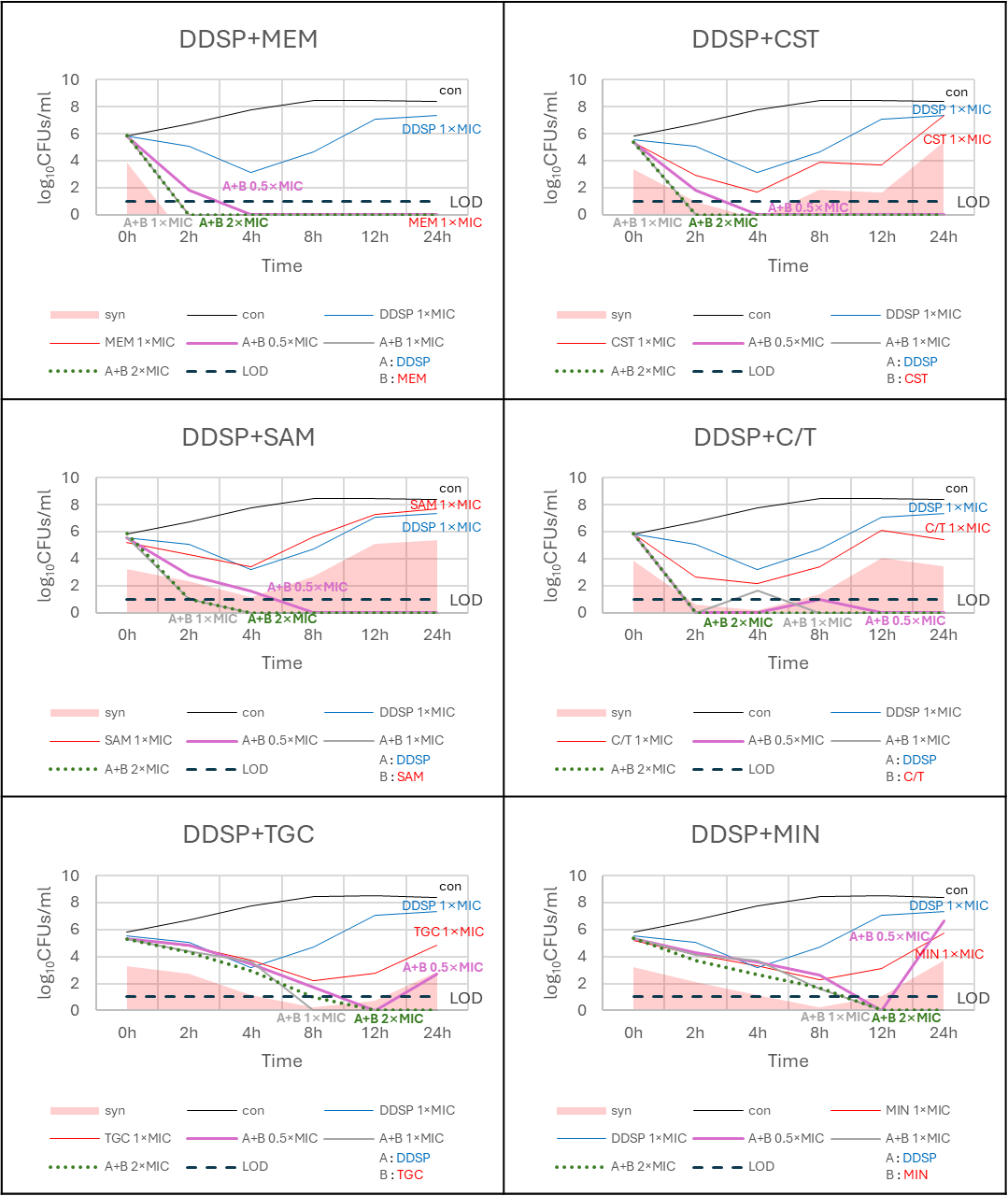


**Supplementary Figure 2.** Time-kill assay results for DD-S052P-based combination regimens against CRAB isolate No. 3. Pink-shaded area indicates the synergistic zone. Each antibiotic was tested alone at 1 × MIC, and in combination at 0.5 × MIC, 1 × MIC, and 2 × MIC. C/T, ceftolozane/tazobactam; CST, colistin; DDSP, DD-S052P; LOD, limit of detection; MEM, meropenem; MIC, minimum inhibitory concentration; MIN, minocycline; SAM, ampicillin/sulbactam; TGC, tigecycline.


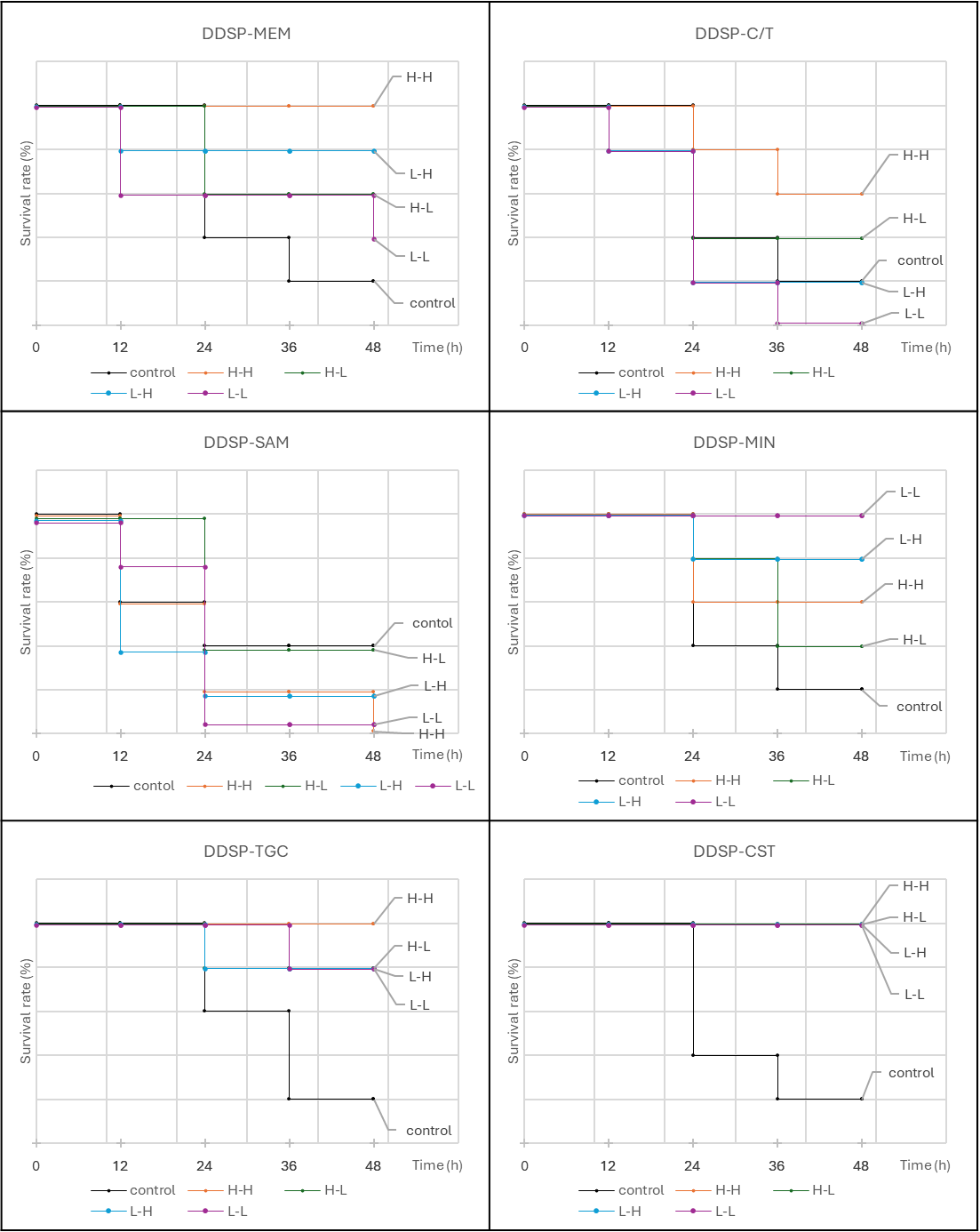


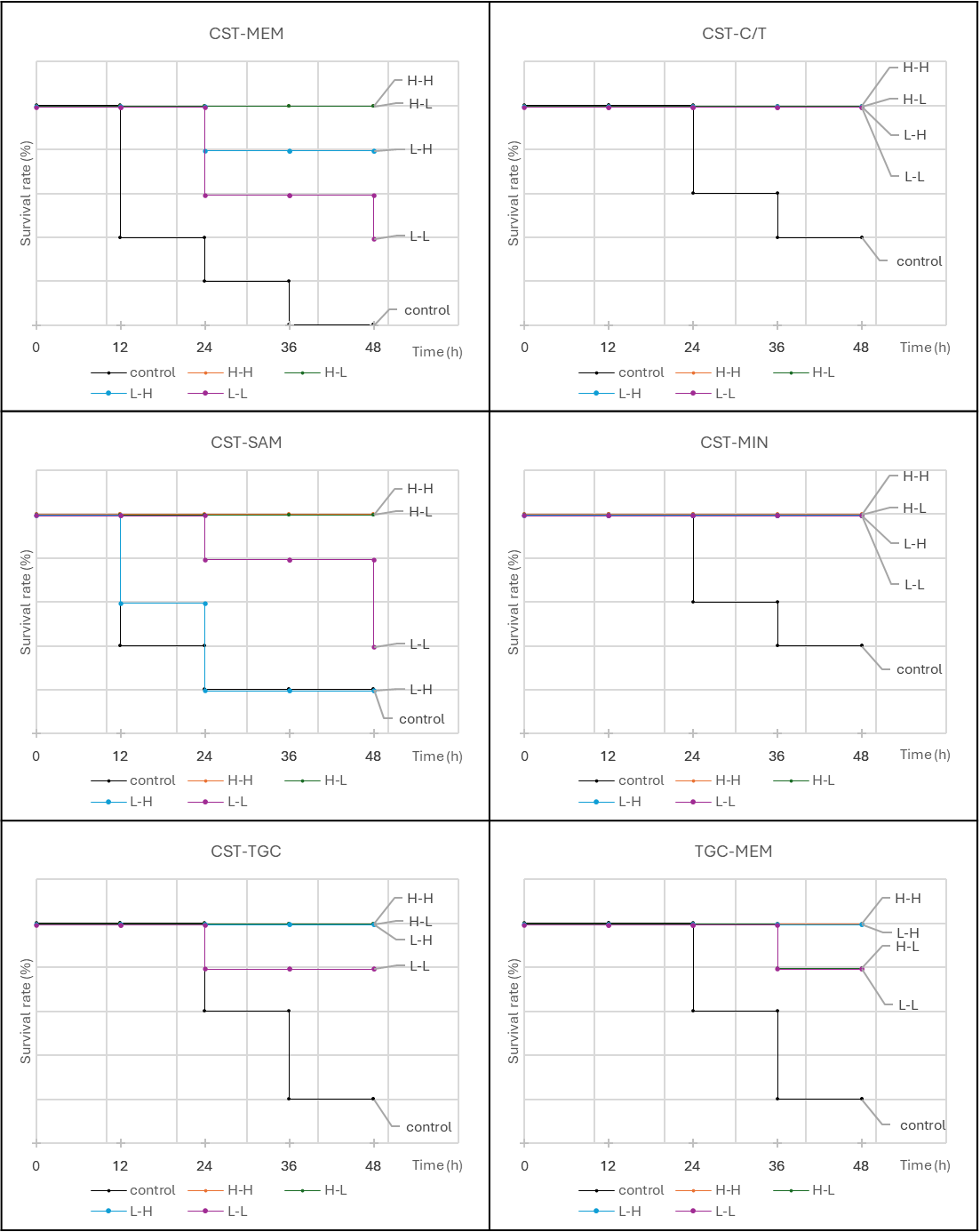


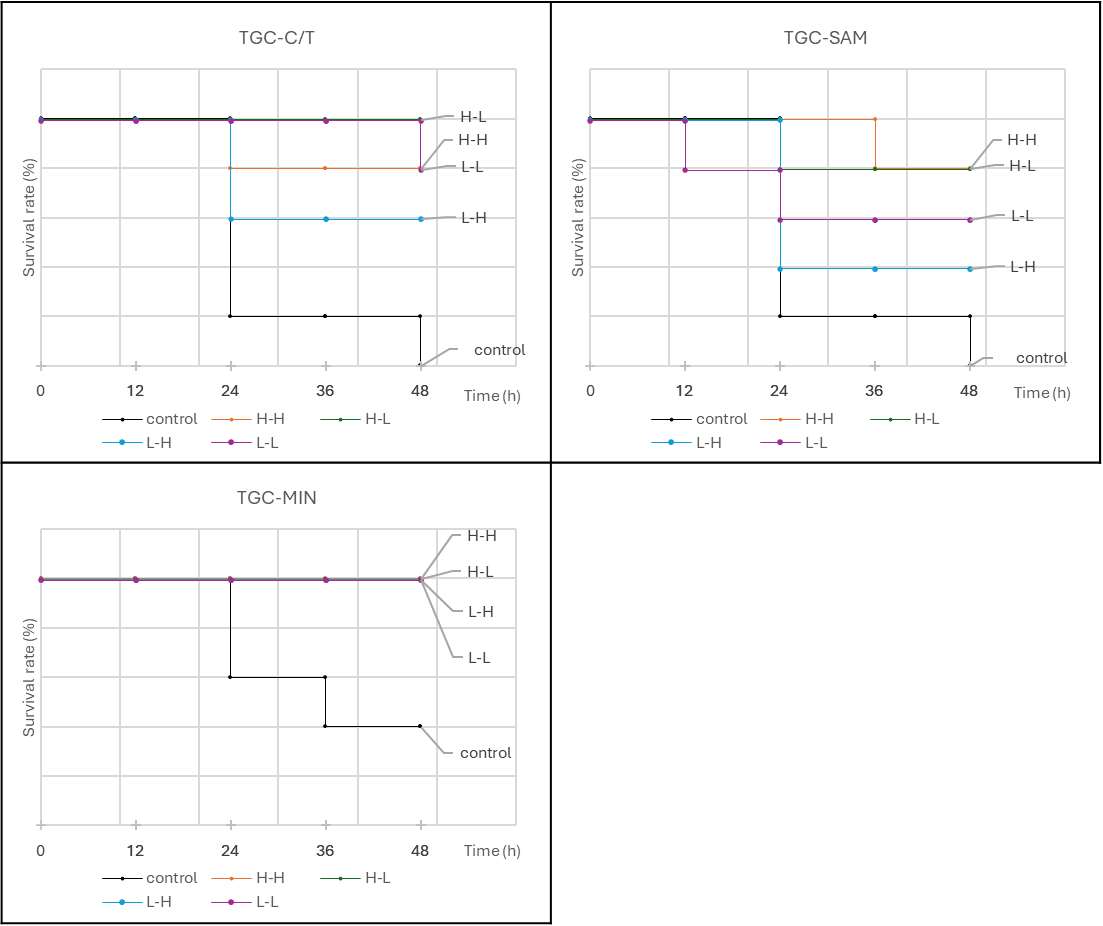


**Supplementary Figure 3**. Kaplan–Meier survival curves of CRAB-infected mice treated with 15 antibiotic combinations. Survival was monitored for 48 h. The curves illustrate the effects of each combination therapy on survival, and differences between the treatment groups were assessed using the log-rank test. N = 5. C/T, ceftolozane/tazobactam; CST, colistin; DDSP, DD-S052P; MEM, meropenem; MIN, minocycline; SAM, ampicillin/sulbactam; TGC, tigecycline.

## Supplementary Tables

**Supplementary Table 1.** MIC Range, MIC₅₀, and MIC₉₀ of clinical isolates

| **Antibiotics**  **(µg/mL)** | **Range** | **MIC_50_** | **MIC_90_** |
| --- | --- | --- | --- |
| MEM | 16–64 | 64 | 64 |
| SAM | 16–128 | 64 | 64 |
| C/T | 1–128 | 32 | 128 |
| CST | 2–16 | 8 | 16 |
| RIF | 2–16 | 2 | 8 |
| MIN | <0.125–4 | 0.5 | 4 |
| PMB | 0.25–0.5 | 0.5 | 0.5 |
| TGC | 0.5–2 | 1 | 2 |
| LVX | 1–255 | 8 | 64 |
| CZA | 2–32 | 32 | 32 |
| TZP | 64–>256 | 256 | >256 |
| DD-S052P | 16–64 | 32 | 64 |

C/T, ceftolozane/tazobactam; CST, colistin; CZA, ceftazidime/avibactam; LVX, levofloxacin; MEM, meropenem; MIC, minimum inhibitory concentration; MIC₅₀, Concentration inhibiting 50% of isolates; MIC₉₀, Concentration inhibiting 90% of isolates; MIN, minocycline; PMB, polymyxin B; RIF, rifampicin; SAM, ampicillin/sulbactam; TGC, tigecycline; TZP, piperacillin/tazobactam.

**Supplementary Table 2.** Representative raw data from the checkerboard assay showing optical density (OD₆₀₀) values for the combination of DD-S052P and ceftolozane/tazobactam against carbapenem-resistant *Acinetobacter baumannii*

| **DDSP**  **C/T** | **0** | **0.25** | **0.5** | **1** | **2** | **4** | **8** | **16** | **32** | **64** | **128** | **256** |
| --- | --- | --- | --- | --- | --- | --- | --- | --- | --- | --- | --- | --- |
| 256 | 0.0417 | 0.0404 | 0.0417 | 0.0412 | 0.0416 | 0.0428 | 0.0425 | 0.0425 | 0.0421 | 0.0424 | 0.0425 | 0.0435 |
| 128 | 0.0385 | 0.0392 | 0.0378 | 0.0382 | 0.039 | 0.0387 | 0.039 | 0.0394 | 0.0416 | 0.0381 | 0.0403 | 0.039 |
| 64 | 0.0367 | 0.0368 | 0.0366 | 0.0361 | 0.037 | 0.0361 | 0.0361 | 0.0371 | 0.0369 | 0.0383 | 0.0385 | 0.0375 |
| 32 | 0.1438 | 0.1404 | 0.1002 | 0.0387 | 0.0362 | 0.0357 | 0.0352 | 0.036 | 0.0361 | 0.0363 | 0.0366 | 0.037 |
| 16 | 0.2861 | 0.26 | 0.2234 | 0.1535 | 0.0362 | 0.0351 | 0.0359 | 0.036 | 0.0362 | 0.0362 | 0.0369 | 0.0371 |
| 8 | 0.3666 | 0.2789 | 0.2403 | 0.1812 | 0.1708 | 0.112 | 0.0357 | 0.0352 | 0.0361 | 0.036 | 0.0464 | 0.0371 |
| 4 | 0.357 | 0.2736 | 0.2315 | 0.2144 | 0.2012 | 0.1671 | 0.111 | 0.0377 | 0.0366 | 0.0389 | 0.0373 | 0.0364 |
| 0 | 0.4447 | 0.3533 | 0.3527 | 0.3077 | 0.324 | 0.2754 | 0.273 | 0.2174 | 0.0383 | 0.0379 | 0.0356 | 0.0377 |

DD-S052P + ceftolozane/tazobactam combination 1

DD-S052P + ceftolozane/tazobactam combination 2

| **DDSP**  **C/T** | **0** | **0.25** | **0.5** | **1** | **2** | **4** | **8** | **16** | **32** | **64** | **128** | **256** |
| --- | --- | --- | --- | --- | --- | --- | --- | --- | --- | --- | --- | --- |
| 256 | 0.0406 | 0.0404 | 0.0422 | 0.0406 | 0.0414 | 0.0424 | 0.0422 | 0.0424 | 0.0407 | 0.0413 | 0.0404 | 0.0418 |
| 128 | 0.0382 | 0.038 | 0.0374 | 0.0375 | 0.0377 | 0.0388 | 0.0388 | 0.0388 | 0.0381 | 0.0381 | 0.0389 | 0.0389 |
| 64 | 0.0366 | 0.0372 | 0.0365 | 0.0358 | 0.0364 | 0.036 | 0.0358 | 0.0372 | 0.0367 | 0.0375 | 0.0377 | 0.038 |
| 32 | 0.1431 | 0.1268 | 0.0871 | 0.0362 | 0.0357 | 0.0351 | 0.0347 | 0.036 | 0.0361 | 0.036 | 0.0364 | 0.0384 |
| 16 | 0.2057 | 0.1685 | 0.1725 | 0.1601 | 0.036 | 0.0348 | 0.036 | 0.0362 | 0.0364 | 0.0365 | 0.0372 | 0.0376 |
| 8 | 0.3037 | 0.1747 | 0.1624 | 0.1522 | 0.1449 | 0.1177 | 0.0356 | 0.0353 | 0.0354 | 0.0359 | 0.0365 | 0.0372 |
| 4 | 0.2623 | 0.1773 | 0.1404 | 0.1266 | 0.1263 | 0.1561 | 0.1193 | 0.0364 | 0.0359 | 0.0358 | 0.0365 | 0.0364 |
| 0 | 0.4414 | 0.2853 | 0.2394 | 0.2558 | 0.1995 | 0.1858 | 0.1905 | 0.1724 | 0.0409 | 0.0355 | 0.0349 | 0.0368 |

| **DDSP**  **C/T** | **0** | **0.25** | **0.5** | **1** | **2** | **4** | **8** | **16** | **32** | **64** | **128** | **256** |
| --- | --- | --- | --- | --- | --- | --- | --- | --- | --- | --- | --- | --- |
| 256 | 0.0427 | 0.0438 | 0.044 | 0.0418 | 0.0437 | 0.044 | 0.0472 | 0.043 | 0.0431 | 0.0421 | 0.0441 | 0.0432 |
| 128 | 0.0433 | 0.04 | 0.0398 | 0.0398 | 0.0401 | 0.0406 | 0.0372 | 0.0413 | 0.038 | 0.04 | 0.0416 | 0.041 |
| 64 | 0.0376 | 0.04 | 0.0401 | 0.037 | 0.0369 | 0.0367 | 0.034 | 0.0385 | 0.0369 | 0.0386 | 0.0379 | 0.0371 |
| 32 | 0.1359 | 0.1193 | 0.1237 | 0.0349 | 0.0356 | 0.0364 | 0.0355 | 0.0381 | 0.0392 | 0.0369 | 0.0369 | 0.0377 |
| 16 | 0.1887 | 0.2788 | 0.1918 | 0.16 | 0.0351 | 0.0338 | 0.0372 | 0.0371 | 0.0373 | 0.0391 | 0.0372 | 0.038 |
| 8 | 0.2172 | 0.1597 | 0.1503 | 0.1574 | 0.1784 | 0.1193 | 0.0388 | 0.0349 | 0.0368 | 0.0373 | 0.0365 | 0.0366 |
| 4 | 0.1952 | 0.1582 | 0.1317 | 0.1291 | 0.1391 | 0.1424 | 0.1226 | 0.0374 | 0.0363 | 0.0374 | 0.037 | 0.0365 |
| 0 | 0.4089 | 0.1972 | 0.1824 | 0.3008 | 0.3009 | 0.1599 | 0.2342 | 0.2068 | 0.0945 | 0.0374 | 0.0366 | 0.0358 |

DD-S052P + ceftolozane/tazobactam combination 3

C/T, ceftolozane/tazobactam; DDSP, DD-S052P.

**Supplementary Table 3**. Results of checkerboard assay for the evaluation of two-drug combinations against clinical isolates of carbapenem-resistant *Acinetobacter baumannii*

| **Clinical isolates** | **MIC of a single agent (mg/L)** | | **MIC in combination (mg/L)** | | **FICI** | **Result of checkerboard assay** |
| --- | --- | --- | --- | --- | --- | --- |
|  | **Drug A** | **Drug B** | **Drug A** | **Drug B** |  |  |
| **DD-S052P + Minocycline** | | | | | | |
| 1 | 4 | 0.031 | 2 | 0.031 | 1.5 | Indifferent |
| 2 | 32 | 0.25 | 2 | 0.031 | 0.188 | Synergistic |
| 3 | 64 | 0.25 | 4 | 0.031 | 0.375 | Synergistic |
| 4 | 64 | 0.5 | 2 | 0.063 | 0.188 | Synergistic |
| 5 | 64 | 4 | 2 | 1 | 0.281 | Synergistic |
| 6 | 64 | 4 | 2 | 1 | 0.281 | Synergistic |
| 7 | 16 | 0.5 | 4 | 0.063 | 0.375 | Synergistic |
| 8 | 32 | 0.25 | 4 | 0.031 | 0.25 | Synergistic |
| 9 | 16 | 0.5 | 2 | 0.063 | 0.375 | Synergistic |
| 10 | 64 | 4 | 2 | 1 | 0.281 | Synergistic |
| 11 | 16 | 1 | 2 | 0.125 | 0.25 | Synergistic |
| 12 | 32 | 0.25 | 4 | 0.031 | 0.375 | Synergistic |
| 13 | 16 | 0.25 | 2 | 0.063 | 0.375 | Synergistic |
| 14 | 32 | 0.5 | 4 | 0.063 | 0.188 | Synergistic |
| 15 | 64 | 0.25 | 4 | 0.063 | 0.313 | Synergistic |
| **DD-S052P + Ampicillin/Sulbactam** | | | | | | |
| 1 | 8 | 8 | 4 | 0.5 | 0.563 | Additive |
| 2 | 32 | 128 | 4 | 16 | 0.25 | Synergistic |
| 3 | 32 | 32 | 4 | 8 | 0.188 | Synergistic |
| 4 | 32 | 64 | 8 | 8 | 0.375 | Synergistic |
| 5 | 32 | 64 | 8 | 8 | 0.375 | Synergistic |
| 6 | 32 | 128 | 8 | 8 | 0.188 | Synergistic |
| 7 | 16 | 64 | 4 | 8 | 0.375 | Synergistic |
| 8 | 32 | 32 | 8 | 4 | 0.375 | Synergistic |
| 9 | 32 | 64 | 4 | 8 | 0.375 | Synergistic |
| 10 | 32 | 64 | 8 | 8 | 0.375 | Synergistic |
| 11 | 16 | 64 | 4 | 16 | 0.5 | Synergistic |
| 12 | 32 | 32 | 8 | 4 | 0.375 | Synergistic |
| 13 | 16 | 32 | 4 | 8 | 0.5 | Synergistic |
| 14 | 32 | 128 | 8 | 16 | 0.258 | Synergistic |
| 15 | 64 | 32 | 8 | 4 | 0.25 | Synergistic |
| **DD-S052P + Meropenem** | | | | | | |
| 1 | 16 | 16 | 4 | 4 | 0.375 | Synergistic |
| 2 | 32 | 128 | 2 | 4 | 0.07 | Synergistic |
| 3 | 64 | 128 | 4 | 16 | 0.188 | Synergistic |
| 4 | 64 | 128 | 0.25 | 4 | 0.047 | Synergistic |
| 5 | 64 | 128 | 4 | 8 | 0.125 | Synergistic |
| 6 | 64 | 128 | 4 | 16 | 0.188 | Synergistic |
| 7 | 16 | 128 | 2 | 32 | 0.375 | Synergistic |
| 8 | 32 | 128 | 4 | 16 | 0.25 | Synergistic |
| 9 | 16 | 128 | 4 | 16 | 0.375 | Synergistic |
| 10 | 64 | 128 | 0.5 | 16 | 0.133 | Synergistic |
| 11 | 16 | 128 | 0.5 | 4 | 0.063 | Synergistic |
| 12 | 32 | 32 | 4 | 8 | 0.375 | Synergistic |
| 13 | 32 | 128 | 2 | 16 | 0.188 | Synergistic |
| 14 | 16 | 32 | 0.25 | 4 | 0.141 | Synergistic |
| 15 | 64 | 32 | 4 | 4 | 0.25 | Synergistic |
| **DD-S052P + Ceftolozane/Tazobactam** | | | | | | |
| 1 | 16 | 1 | 4 | 0.5 | 0.75 | Additive |
| 2 | 16 | 16 | 4 | 1 | 0.266 | Synergistic |
| 3 | 64 | 16 | 4 | 4 | 0.313 | Synergistic |
| 4 | 64 | 16 | 8 | 2 | 0.25 | Synergistic |
| 5 | 64 | 32 | 8 | 4 | 0.313 | Synergistic |
| 6 | 64 | 32 | 16 | 2 | 0.375 | Synergistic |
| 7 | 16 | 16 | 4 | 4 | 0.5 | Synergistic |
| 8 | 16 | 64 | 4 | 16 | 0.5 | Synergistic |
| 9 | 16 | 8 | 8 | 0.25 | 0.625 | Additive |
| 10 | 64 | 64 | 8 | 4 | 0.25 | Synergistic |
| 11 | 16 | 8 | 4 | 2 | 0.531 | Additive |
| 12 | 32 | 128 | 8 | 8 | 0.313 | Synergistic |
| 13 | 32 | 64 | 8 | 8 | 0.375 | Synergistic |
| 14 | 16 | 128 | 4 | 0.25 | 0.252 | Synergistic |
| 15 | 64 | 8 | 4 | 0.5 | 0.188 | Synergistic |
| **DD-S052P + Tigecycline** | | | | | | |
| 1 | 16 | 0.5 | 4 | 0.25 | 0.75 | Additive |
| 2 | 64 | 1 | 8 | 0.5 | 0.625 | Additive |
| 3 | 64 | 1 | 32 | 0.5 | 1 | Additive |
| 4 | 64 | 1 | 32 | 0.031 | 0.563 | Additive |
| 5 | 64 | 0.5 | 4 | 0.125 | 0.313 | Synergistic |
| 6 | 64 | 1 | 16 | 0.5 | 0.75 | Additive |
| 7 | 16 | 0.5 | 8 | 0.063 | 0.75 | Additive |
| 8 | 32 | 0.5 | 16 | 0.063 | 0.625 | Additive |
| 9 | 16 | 0.5 | 16 | 0.031 | 1.063 | Indifferent |
| 10 | 64 | 1 | 8 | 0.063 | 0.188 | Synergistic |
| 11 | 16 | 1 | 4 | 0.5 | 0.625 | Additive |
| 12 | 32 | 1 | 16 | 0.125 | 0.625 | Additive |
| 13 | 32 | 0.5 | 16 | 0.031 | 0.563 | Additive |
| 14 | 32 | 0.5 | 16 | 0.063 | 0.563 | Additive |
| 15 | 64 | 1 | 32 | 0.125 | 0.625 | Additive |
| **DD-S052P + Colistin** | | | | | | |
| 1 | 16 | 32 | 8 | 8 | 0.5 | Synergistic |
| 2 | 32 | 16 | 8 | 0.5 | 0.281 | Synergistic |
| 3 | 64 | 16 | 16 | 4 | 0.5 | Synergistic |
| 4 | 64 | 8 | 16 | 0.5 | 0.313 | Synergistic |
| 5 | 64 | 2 | 16 | 0.25 | 0.313 | Synergistic |
| 6 | 64 | 4 | 16 | 0.5 | 0.313 | Synergistic |
| 7 | 16 | 8 | 4 | 1 | 0.563 | Additive |
| 8 | 32 | 4 | 8 | 0.25 | 0.313 | Synergistic |
| 9 | 16 | 16 | 8 | 2 | 0.625 | Additive |
| 10 | 64 | 2 | 4 | 0.5 | 0.313 | Synergistic |
| 11 | 16 | 8 | 8 | 4 | 0.75 | Additive |
| 12 | 32 | 4 | 8 | 0.5 | 0.25 | Synergistic |
| 13 | 32 | 4 | 8 | 0.25 | 0.25 | Synergistic |
| 14 | 32 | 8 | 4 | 1 | 0.188 | Synergistic |
| 15 | 64 | 16 | 16 | 1 | 0.281 | Synergistic |
| **Colistin + Minocycline** | | | | | | |
| 1 | 32 | 0.125 | 8 | 0.031 | 0.5 | Synergistic |
| 2 | 16 | 1 | 4 | 0.5 | 1.063 | Indifferent |
| 3 | 16 | 0.25 | 8 | 0.125 | 1 | Additive |
| 4 | 32 | 0.5 | 4 | 0.25 | 0.75 | Additive |
| 5 | 4 | 4 | 1 | 0.031 | 0.258 | Synergistic |
| 6 | 2 | 4 | 1 | 2 | 1 | Additive |
| 7 | 32 | 0.5 | 16 | 0.25 | 1 | Additive |
| 8 | 8 | 1 | 4 | 0.031 | 0.75 | Additive |
| 9 | 16 | 1 | 8 | 0.031 | 0.75 | Additive |
| 10 | 8 | 4 | 4 | 0.25 | 2.125 | Antagonism |
| 11 | 16 | 1 | 4 | 0.5 | 0.625 | Additive |
| 12 | 4 | 1 | 0.5 | 1 | 1.125 | Indifferent |
| 13 | 8 | 0.5 | 2 | 0.25 | 0.75 | Additive |
| 14 | 16 | 0.5 | 2 | 0.25 | 0.75 | Additive |
| 15 | 32 | 0.5 | 8 | 0.125 | 0.625 | Additive |
| **Colistin + Ampicillin/Sulbactam** | | | | | | |
| 1 | 16 | 32 | 0.063 | 16 | 0.502 | Additive |
| 2 | 8 | 64 | 0.063 | 64 | 1.004 | Indifferent |
| 3 | 16 | 64 | 0.5 | 32 | 0.531 | Additive |
| 4 | 16 | 64 | 0.063 | 32 | 0.504 | Additive |
| 5 | 2 | 128 | 0.25 | 64 | 0.531 | Additive |
| 6 | 2 | 128 | 0.5 | 64 | 0.625 | Additive |
| 7 | 16 | 64 | 0.063 | 32 | 0.504 | Additive |
| 8 | 4 | 64 | 1 | 32 | 1 | Additive |
| 9 | 16 | 64 | 8 | 32 | 0.625 | Additive |
| 10 | 8 | 64 | 0.5 | 32 | 0.563 | Additive |
| 11 | 16 | 64 | 2 | 32 | 0.563 | Additive |
| 12 | 8 | 64 | 2 | 16 | 0.5 | Synergistic |
| 13 | 8 | 32 | 2 | 4 | 0.375 | Synergistic |
| 14 | 8 | 128 | 2 | 64 | 1 | Additive |
| 15 | 16 | 32 | 4 | 16 | 0.625 | Additive |
| **Colistin + Ceftolozane/Tazobactam** | | | | | | |
| 1 | 16 | 2 | 8 | 1 | 0.75 | Additive |
| 2 | 16 | 16 | 0.063 | 16 | 1.004 | Indifferent |
| 3 | 16 | 16 | 8 | 8 | 1 | Additive |
| 4 | 8 | 32 | 0.5 | 16 | 0.625 | Additive |
| 5 | 4 | 64 | 0.5 | 4 | 0.25 | Synergistic |
| 6 | 2 | 32 | 0.5 | 8 | 0.5 | Synergistic |
| 7 | 16 | 8 | 4 | 4 | 0.75 | Additive |
| 8 | 4 | 128 | 0.25 | 64 | 0.563 | Additive |
| 9 | 16 | 8 | 0.063 | 8 | 1.004 | Indifferent |
| 10 | 8 | 32 | 4 | 16 | 1 | Additive |
| 11 | 16 | 8 | 0.063 | 8 | 1.004 | Indifferent |
| 12 | 4 | 128 | 1 | 64 | 0.75 | Additive |
| 13 | 4 | 128 | 0.5 | 64 | 0.625 | Additive |
| 14 | 8 | 128 | 0.063 | 128 | 1.008 | Indifferent |
| 15 | 16 | 16 | 2 | 8 | 0.563 | Additive |
| **Colistin + Meropenem** | | | | | | |
| 1 | 16 | 16 | 0.125 | 16 | 1.008 | Indifferent |
| 2 | 16 | 128 | 0.5 | 64 | 0.516 | Additive |
| 3 | 16 | 64 | 8 | 8 | 0.625 | Additive |
| 4 | 8 | 64 | 2 | 32 | 1 | Additive |
| 5 | 2 | 128 | 0.25 | 32 | 0.25 | Synergistic |
| 6 | 2 | 128 | 0.5 | 32 | 0.375 | Synergistic |
| 7 | 8 | 64 | 0.5 | 32 | 0.563 | Additive |
| 8 | 4 | 128 | 0.063 | 64 | 0.516 | Additive |
| 9 | 16 | 128 | 0.125 | 64 | 0.504 | Additive |
| 10 | 8 | 64 | 2 | 32 | 0.563 | Additive |
| 11 | 16 | 64 | 8 | 16 | 0.75 | Additive |
| 12 | 4 | 64 | 0.5 | 32 | 0.75 | Additive |
| 13 | 4 | 64 | 1 | 32 | 0.75 | Additive |
| 14 | 16 | 32 | 1 | 16 | 0.531 | Additive |
| 15 | 8 | 32 | 4 | 8 | 0.75 | Additive |
| **Colistin + Tigecycline** | | | | | | |
| 1 | 32 | 0.5 | 0.031 | 0.25 | 0.501 | Additive |
| 2 | 16 | 1 | 32 | 0.5 | 2.25 | Antagonism |
| 3 | 16 | 1 | 32 | 0.5 | 2.5 | Antagonism |
| 4 | 8 | 1 | 32 | 0.25 | 2.25 | Antagonism |
| 5 | 2 | 1 | 4 | 0.5 | 2.5 | Antagonism |
| 6 | 2 | 2 | 4 | 1 | 2.25 | Antagonism |
| 7 | 8 | 1 | 32 | 0.25 | 8.125 | Antagonism |
| 8 | 4 | 1 | 16 | 0.125 | 2.125 | Antagonism |
| 9 | 16 | 1 | 32 | 0.25 | 2.25 | Antagonism |
| 10 | 8 | 1 | 4 | 1 | 1.004 | Indifferent |
| 11 | 16 | 1 | 32 | 0.25 | 2.25 | Antagonism |
| 12 | 4 | 1 | 16 | 0.25 | 4.125 | Antagonism |
| 13 | 4 | 0.5 | 8 | 0.25 | 2.25 | Antagonism |
| 14 | 16 | 0.5 | 0.031 | 0.5 | 1.002 | Indifferent |
| 15 | 16 | 1 | 32 | 0.25 | 2.25 | Antagonism |
| **Tigecycline + Meropenem** | | | | | | |
| 1 | 0.5 | 16 | 0.125 | 8 | 0.75 | Additive |
| 2 | 1 | 64 | 0.031 | 64 | 1.031 | Indifferent |
| 3 | 1 | 64 | 0.5 | 32 | 0.75 | Additive |
| 4 | 1 | 64 | 0.125 | 32 | 0.531 | Additive |
| 5 | 1 | 128 | 0.5 | 32 | 1 | Additive |
| 6 | 1 | 128 | 0.125 | 64 | 0.625 | Additive |
| 7 | 1 | 64 | 0.5 | 32 | 0.75 | Additive |
| 8 | 0.5 | 64 | 0.031 | 64 | 1.063 | Indifferent |
| 9 | 0.5 | 64 | 0.031 | 64 | 1.063 | Indifferent |
| 10 | 2 | 64 | 1 | 4 | 0.563 | Additive |
| 11 | 1 | 64 | 0.5 | 32 | 1 | Additive |
| 12 | 1 | 64 | 0.25 | 32 | 0.75 | Additive |
| 13 | 1 | 64 | 0.125 | 32 | 0.75 | Additive |
| 14 | 0.5 | 32 | 0.125 | 16 | 0.75 | Additive |
| 15 | 1 | 32 | 0.5 | 16 | 1 | Additive |
| **Tigecycline + Ceftolozane/Tazobactam** | | | | | | |
| 1 | 0.5 | 2 | 0.5 | 0.25 | 1.125 | Indifferent |
| 2 | 1 | 32 | 0.125 | 16 | 0.625 | Additive |
| 3 | 1 | 16 | 1 | 4 | 1.016 | Indifferent |
| 4 | 1 | 32 | 0.5 | 16 | 1 | Additive |
| 5 | 1 | 64 | 0.125 | 32 | 0.563 | Additive |
| 6 | 2 | 64 | 1 | 0.25 | 0.625 | Additive |
| 7 | 1 | 16 | 0.25 | 8 | 0.75 | Additive |
| 8 | 1 | 128 | 0.5 | 32 | 0.75 | Additive |
| 9 | 1 | 16 | 0.5 | 4 | 0.75 | Additive |
| 10 | 1 | 64 | 2 | 4 | 2.008 | Antagonism |
| 11 | 1 | 8 | 0.5 | 4 | 1 | Additive |
| 12 | 1 | 128 | 0.5 | 64 | 1 | Additive |
| 13 | 1 | 128 | 0.5 | 32 | 0.75 | Additive |
| 14 | 1 | 128 | 0.5 | 64 | 1 | Additive |
| 15 | 1 | 16 | 0.5 | 8 | 1 | Additive |
| **Tigecycline + Ampicillin/Sulbactam** | | | | | | |
| 1 | 1 | 16 | 0.5 | 0.25 | 0.516 | Additive |
| 2 | 2 | 64 | 1 | 1 | 0.516 | Additive |
| 3 | 2 | 64 | 0.125 | 32 | 0.625 | Additive |
| 4 | 1 | 32 | 0.5 | 16 | 1 | Additive |
| 5 | 2 | 128 | 0.25 | 32 | 0.375 | Synergistic |
| 6 | 2 | 128 | 0.125 | 64 | 0.563 | Additive |
| 7 | 1 | 32 | 0.5 | 16 | 1 | Additive |
| 8 | 1 | 64 | 0.5 | 16 | 0.75 | Additive |
| 9 | 1 | 64 | 0.5 | 16 | 0.75 | Additive |
| 10 | 2 | 32 | 0.125 | 16 | 0.563 | Additive |
| 11 | 1 | 64 | 0.5 | 32 | 1 | Additive |
| 12 | 1 | 64 | 0.5 | 16 | 0.75 | Additive |
| 13 | 1 | 32 | 0.5 | 8 | 1 | Additive |
| 14 | 1 | 128 | 1 | 0.25 | 1.008 | Indifferent |
| 15 | 1 | 32 | 2 | 2 | 2.063 | Antagonism |
| **Tigecycline + Minocycline** | | | | | | |
| 1 | 0.5 | 0.125 | 0.125 | 0.031 | 0.516 | Synergistic |
| 2 | 1 | 0.5 | 0.5 | 0.125 | 0.516 | Additive |
| 3 | 2 | 1 | 0.5 | 0.25 | 0.625 | Additive |
| 4 | 1 | 0.5 | 0.5 | 0.25 | 1 | Additive |
| 5 | 1 | 4 | 0.25 | 0.063 | 0.375 | Synergistic |
| 6 | 1 | 4 | 0.125 | 4 | 0.563 | Indifferent |
| 7 | 1 | 1 | 0.5 | 0.063 | 1 | Additive |
| 8 | 0.5 | 1 | 0.25 | 0.25 | 0.75 | Additive |
| 9 | 1 | 1 | 0.5 | 0.25 | 0.75 | Additive |
| 10 | 1 | 4 | 0.5 | 1 | 0.563 | Additive |
| 11 | 1 | 2 | 0.125 | 1 | 1 | Additive |
| 12 | 1 | 1 | 0.5 | 0.063 | 0.75 | Additive |
| 13 | 0.5 | 1 | 0.25 | 0.25 | 1 | Additive |
| 14 | 0.5 | 1 | 0.25 | 0.5 | 1.008 | Additive |
| 15 | 1 | 0.5 | 0.5 | 0.25 | 2.063 | Additive |

MIC, minimum inhibitory concentration; FICI, fractional inhibitory concentration index (an indicator of interaction between two antibiotics).

**Supplementary Table 4.** Results of time-kill assay showing colony-forming units for the evaluation of two-drug combinations against clinical isolates of carbapenem-resistant *Acinetobacter baumannii*

| **DD-S052P + ampicillin/sulbactam** | | | | | | |
| --- | --- | --- | --- | --- | --- | --- |
|  | DDSP 1X | SAM 1X | DDSP + SAM 0.5X | DDSP + SAM 1X | DDSP + SAM 2X | |
| 0 h | 365000 | 158750 | 345000 | 390000 | 680000 | |
| 2 h | 110000 | 20850 | 550 | 10 | 10 | |
| 4 h | 1430 | 2360 | 40 | 0 | 0 | |
| 8 h | 47950 | 415000 | 0 | 0 | 0 | |
| 12 h | 12250000 | 19100000 | 0 | 0 | 0 | |
| 24 h | 22350000 | 53100000 | 0 | 0 | 0 | |
| **DD-S052P + ceftolozane/tazobactam** | | | | | | |
|  | DDSP 1X | C/T 1X | DDSP + C/T 0.5X | DDSP + C/T 1X | DDSP + C/T 2X | |
| 0 h | 680000 | 680000 | 680000 | 680000 | 680000 | |
| 2 h | 110000 | 420 | 0 | 0 | 0 | |
| 4 h | 1430 | 140 | 0 | 40 | 0 | |
| 8 h | 47950 | 2410 | 10 | 0 | 0 | |
| 12 h | 12250000 | 1210000 | 0 | 0 | 0 | |
| 24 h | 22350000 | 250000 | 0 | 0 | 0 | |
| **DD-S052P + minocycline** | | | | | | |
|  | DDSP 1X | MIN 1X | DDSP + MIN 0.5X | DDSP + MIN 1X | DDSP + MIN 2X | |
| 0 h | 365000 | 167500 | 223000 | 223000 | 223000 | |
| 2 h | 110000 | 13250 | 20700 | 12350 | 4835 | |
| 4 h | 1430 | 2125 | 4215 | 5270 | 455 | |
| 8 h | 47950 | 180 | 410 | 40 | 50 | |
| 12 h | 12250000 | 1220 | 0 | 0 | 0 | |
| 24 h | 22350000 | 520000 | 4550000 | 0 | 0 | |
| **DD-S052P + meropenem** | | | | | | |
|  | DDSP 1X | MEM 1X | DDSP + SAM 0.5X | DDSP + SAM 1X | DDSP + SAM 2X | |
| 0 h | 680000 | 680000 | 680000 | 680000 | 680000 | |
| 2 h | 110000 | 0 | 60 | 0 | 0 | |
| 4 h | 1430 | 0 | 0 | 0 | 0 | |
| 8 h | 47950 | 0 | 0 | 0 | 0 | |
| 12 h | 12250000 | 0 | 0 | 0 | 0 | |
| 24 h | 22350000 | 0 | 0 | 0 | 0 | |
| **DD-S052P + tigecycline** | | | | | | |
|  | DDSP 1X | TGC 1X | DDSP + TGC 0.5X | DDSP + TGC 1X | DDSP + TGC 2X | |
| 0 h | 365000 | 200000 | 200000 | 200000 | 200000 | |
| 2 h | 110000 | 57950 | 70050 | 23600 | 20550 | |
| 4 h | 1430 | 5585 | 2885 | 4335 | 840 | |
| 8 h | 47950 | 160 | 55 | 0 | 10 | |
| 12 h | 12250000 | 550 | 0 | 0 | 0 | |
| 24 h | 22350000 | 70000 | 470 | 0 | 0 | |
| **DD-S052P + colistin** | | | | | | |
|  | DDSP 1X | CST 1X | DDSP + CST 0.5X | DDSP + CST 1X | DDSP + CST 2X | |
| 0 h | 365000 | 223000 | 223000 | 223000 | 223000 | |
| 2 h | 110000 | 885 | 65 | 0 | 0 | |
| 4 h | 1430 | 50 | 0 | 0 | 0 | |
| 8 h | 47950 | 7550 | 0 | 0 | 0 | |
| 12 h | 12250000 | 4455 | 0 | 0 | 0 | |
| 24 h | 22350000 | 22000000 | 0 | 0 | 0 | |
| **Colistin + ampicillin/sulbactam** | | | | | | |
|  | CST 1X | SAM 1X | CST + SAM 0.5X | CST + SAM 1X | CST + SAM 2X | |
| 0 h | 223000 | 158750 | 308500 | 308500 | 308500 | |
| 2 h | 885 | 20850 | 10 | 0 | 0 | |
| 4 h | 50 | 2360 | 0 | 0 | 0 | |
| 8 h | 7550 | 415000 | 4695 | 0 | 0 | |
| 12 h | 4455 | 19100000 | 13875 | 0 | 0 | |
| 24 h | 22000000 | 53100000 | 21750 | 0 | 0 | |
| **Colistin + ceftolozane/tazobactam** | | | | | | |
|  | CST 1X | C/T 1X | CST + C/T 0.5X | CST + C/T 1X | CST + C/T 2X |  |
| 0 h | 223000 | 680000 | 308500 | 308500 | 308500 |  |
| 2 h | 885 | 420 | 20 | 0 | 0 |  |
| 4 h | 50 | 140 | 20 | 0 | 0 |  |
| 8 h | 7550 | 2410 | 1850 | 0 | 0 |  |
| 12 h | 4455 | 1210000 | 45300 | 0 | 0 |  |
| 24 h | 22000000 | 250000 | 35350000 | 0 | 0 |  |
| **Colistin + minocycline** | | | | | | |
|  | CST 1X | MIN 1X | CST + MIN 0.5X | CST + MIN 1X | CST + MIN 2X |  |
| 0 h | 223000 | 167500 | 223000 | 223000 | 223000 |  |
| 2 h | 885 | 13250 | 185 | 160 | 250 |  |
| 4 h | 50 | 2125 | 10 | 0 | 0 |  |
| 8 h | 7550 | 180 | 0 | 0 | 0 |  |
| 12 h | 4455 | 1220 | 0 | 0 | 0 |  |
| 24 h | 22000000 | 520000 | 0 | 0 | 0 |  |
| **Colistin + meropenem** | | | | | | |
|  | CST 1X | MEM 1X | MEM + CST 0.5X | MEM + CST 1X | MEM + CST 2X |  |
| 0 h | 223000 | 680000 | 223000 | 223000 | 223000 |  |
| 2 h | 885 | 0 | 335 | 240 | 595 |  |
| 4 h | 50 | 0 | 0 | 0 | 0 |  |
| 8 h | 7550 | 0 | 0 | 0 | 0 |  |
| 12 h | 4455 | 0 | 440 | 0 | 0 |  |
| 24 h | 22000000 | 0 | 3765 | 0 | 0 |  |
| **Colistin + tigecycline** | | | | | | |
|  | CST 1X | TGC 1X | CST + TGC 0.5X | CST + TGC 1X | CST + TGC 2X |  |
| 0 h | 223000 | 200000 | 223000 | 223000 | 223000 |  |
| 2 h | 885 | 57950 | 1410 | 1055 | 120 |  |
| 4 h | 50 | 5585 | 110 | 20 | 0 |  |
| 8 h | 7550 | 160 | 0 | 0 | 0 |  |
| 12 h | 4455 | 550 | 20 | 0 | 0 |  |
| 24 h | 22000000 | 70000 | 515 | 0 | 0 |  |
| **Tigecycline + ampicillin/sulbactam** | | | | | | |
|  | TGC 1X | SAM 1X | TGC + SAM 0.5X | TGC + SAM 1X | TGC + SAM 2X |  |
| 0 h | 200000 | 158750 | 200000 | 200000 | 200000 |  |
| 2 h | 57950 | 20850 | 85000 | 105000 | 21900 |  |
| 4 h | 5585 | 2360 | 2945 | 2400 | 1770 |  |
| 8 h | 160 | 415000 | 130 | 160 | 510 |  |
| 12 h | 550 | 19100000 | 190 | 215 | 50 |  |
| 24 h | 70000 | 53100000 | 10600 | 360 | 55 |  |
| **Tigecycline + ceftolozane/tazobactam** | | | | | | |
|  | TGC 1X | C/T 1X | TGC + C/T 0.5X | TGC + C/T 1X | TGC + C/T 2X |  |
| 0 h | 200000 | 680000 | 200000 | 200000 | 200000 |  |
| 2 h | 57950 | 420 | 62250 | 85100 | 2325 |  |
| 4 h | 5585 | 140 | 1985 | 6300 | 625 |  |
| 8 h | 160 | 2410 | 10 | 175 | 65 |  |
| 12 h | 550 | 1210000 | 70 | 0 | 20 |  |
| 24 h | 70000 | 250000 | 110 | 0 | 0 |  |
| **Tigecycline + minocycline** | | | | | | |
|  | TGC 1X | MIN 1X | TGC + MIN 0.5X | TGC + MIN 1X | TGC + MIN 2X |  |
| 0 h | 200000 | 167500 | 223000 | 223000 | 223000 |  |
| 2 h | 57950 | 13250 | 4765 | 3020 | 6250 |  |
| 4 h | 5585 | 2125 | 2240 | 3460 | 4165 |  |
| 8 h | 160 | 180 | 1070 | 1205 | 1995 |  |
| 12 h | 550 | 1220 | 635 | 595 | 805 |  |
| 24 h | 70000 | 520000 | 1695000 | 20 | 255 |  |
| **Tigecycline + meropenem** | | | | | | |
|  | TGC 1X | MEM 1X | TGC + MEM 0.5X | TGC + MEM 1X | TGC + MEM 2X |  |
| 0 h | 200000 | 680000 | 308500 | 308500 | 308500 |  |
| 2 h | 57950 | 0 | 1320 | 455 | 850 |  |
| 4 h | 5585 | 0 | 2745 | 830 | 690 |  |
| 8 h | 160 | 0 | 215 | 90 | 30 |  |
| 12 h | 550 | 0 | 1745 | 0 | 0 |  |
| 24 h | 70000 | 0 | 7855 | 0 | 0 |  |

CST, colistin; C/T, ceftolozane/tazobactam; DDSP, DD-S052P; MEM, meropenem; MIC, minimum inhibitory concentration; MIN, minocycline; SAM, ampicillin/sulbactam; TGC, tigecycline.

**Supplementary Table 5**. Results of time-kill assay for monotherapy (1 × MIC)

|  | 2 h | 4 h | 8 h | 12 h | 24 h |
| --- | --- | --- | --- | --- | --- |
| DDSP |  |  |  |  |  |
| C/T |  |  |  |  |  |
| MEM |  |  |  |  |  |
| SAM |  |  |  |  |  |
| TGC |  |  |  |  |  |
| CST |  |  |  |  |  |
| MIN |  |  |  |  |  |

Black, gray, and white cells indicate bactericidal activity, bacteriostatic activity, and bacterial growth, respectively.

Bactericidal activity: ≥ 3 log_10_ (at least 1,000-fold) reduction from the initial inoculum at 24 h.

Bacteriostatic activity: <3 log_10_ reduction to no change from the initial inoculum at 24 h.

C/T, ceftolozane/tazobactam; CST, colistin; DDSP, DD-S052P; MEM, meropenem; MIC, minimum inhibitory concentration; MIN, minocycline; SAM, ampicillin/sulbactam; TGC, tigecycline.

**Supplementary Table 6**. Antibiotic doses used in animal experiments

| **Dose** | **DDSP** | **MEM** | **C/T** | **TGC** | **CST** | **MIN** | **SAM** |
| --- | --- | --- | --- | --- | --- | --- | --- |
| High (H) | 10 mg/kg | 40 mg/kg | 10 mg/kg | 0.5 mg/kg | 10 mg/kg | 0.5 mg/kg | 40 mg/kg |
| Low (L) | 2.5 mg/kg | 10 mg/kg | 2.5 mg/kg | 0.125 mg/kg | 2.5 mg/kg | 0.125 mg/kg | 10 mg/kg |

C/T, ceftolozane/tazobactam; CST, colistin; DDSP, DD-S052P; MEM, meropenem; MIN, minocycline; SAM, ampicillin/sulbactam; TGC, tigecycline.
